# Spatial learning strengthens hippocampal but not prefrontal influence on nucleus reuniens in rats

**DOI:** 10.3389/fnbeh.2026.1939794

**Published:** 2026-09-25

**Authors:** Tristan Baumann, Oxana Eschenko

**Affiliations:** Body-Brain Cybernetics, Max Planck Institute for Biological Cybernetics, Tübingen, Germany

**Keywords:** cross-regional communication, information flow, neural network dynamics, neural oscillations, nucleus reuniens, spatial learning

## Abstract

**Introduction:**

Coordinated interactions between the hippocampus (HC) and prefrontal cortex (PFC) that are essential for spatial navigation occur despite relatively asymmetric anatomical connectivity. The midline thalamic nucleus reuniens (RE) is reciprocally connected with both regions and has been proposed to support HC-PFC communication.

**Methods:**

Here, we examined local and long-range neural dynamics within the HC-RE-PFC circuit while adult male rats learned to navigate a complex maze across multiple days. Using spectral power, coherence, and Granger causality analyses within linear mixed-effects models to control for locomotion.

**Results:**

We observed learning-related decreases in theta power across all regions, despite increased velocity with better task performance. In contrast, beta (15–30 Hz) increased in HC but decreased in RE and PFC. Notably, HC-to-RE information flow in theta, beta, and low-gamma bands increased with learning, while RE-to-HC influences decreased. Direct HC-PFC interactions remained comparatively stable, and PFC-RE beta coherence decreased with learning.

**Discussion:**

These findings reveal a dynamic reconfiguration of HC-RE-PFC network interactions during spatial learning, driven primarily by a selective increase in hippocampal, but not prefrontal, influence on RE. The directional asymmetry between HC-RE and RE-PFC pathways is inconsistent with a simple relay account and instead supports a role for RE as an active participant in spatial cognition.

## Highlights

- Spatial learning strengthens hippocampal-reuniens functional connectivity
- Spatial learning increases HC-to-RE information flow
- Beta-band HC-RE interactions showed the strongest learning-related effects
- PFC-RE beta synchrony decreased as navigation became more efficient

## Introduction

Spatial navigation is supported by coordinated activity within a distributed brain network, with the hippocampus (HC) and prefrontal cortex (PFC) serving as key hubs (Granon and Poucet, 1995; Colgin, 2011; Ito et al., 2015; Ito, 2018). The HC rapidly encodes spatial information and temporally stabilizes new memory traces (Preston and Eichenbaum, 2013; Jayachandran et al., 2023), whereas the PFC contributes to contextual representation, selective memory retrieval, and action planning (Xu and Südhof, 2013; Eichenbaum, 2017). Furthermore, prefrontal-hippocampal interactions are essential for many aspects of spatial cognition, such as spatial working memory, decision-making, and memory consolidation (Buzsáki, 1996; Colgin, 2011; Eichenbaum, 2017). In rodents, disruptions in HC-PFC communication lead to substantial deficits in spatial learning and memory (Xu and Südhof, 2013; Lopez et al., 2012; Wirt and Hyman, 2017). Despite strong evidence for functional interactions, anatomical connectivity between HC and PFC is notably asymmetric, with HC receiving limited direct input from PFC (Swanson, 1981; Vertes, 2006; Wirt and Hyman, 2017; Rajasethupathy et al., 2015). This anatomical imbalance underscores the significance of indirect pathways in mediating bidirectional HC-PFC communication, and raises the question of which intermediary structures are functionally involved.

The midline thalamic nucleus reuniens (RE) is anatomically well-positioned to mediate communication between HC and PFC. RE receives input from deep layers of the prelimbic and infralimbic cortices and projects back to both superficial and deep layers (Scheel et al., 2020; McKenna and Vertes, 2004; Vertes et al., 2006). It is also densely innervated by ventral HC and subiculum and projects to the stratum lacunosum-moleculare of ventral CA1–a layer containing distal dendrites of excitatory and inhibitory neurons (Scheel et al., 2020; Varela et al., 2014; Vertes et al., 2015; Mathiasen et al., 2020). Optogenetic activation of RE evokes excitatory responses in CA1 (Hauer et al., 2019), and a subset of RE neurons sends collaterals to both HC and PFC (Hoover and Vertes, 2012; Varela et al., 2014), providing a potential substrate for simultaneous modulation of both regions.

Functionally, RE has been implicated in a range of spatial navigation and memory processes. Lesions or inactivation of RE lead to deficits in spatial working memory (Davoodi et al., 2009; Hallock et al., 2016; Hembrook and Mair, 2011; Hembrook et al., 2012; Layfield et al., 2015; Maisson et al., 2018; Viena et al., 2018), consolidation (Loureiro et al., 2012; Xu and Südhof, 2013), and retrieval (Mei et al., 2018; Barker and Warburton, 2018). In rodents, RE neurons show trajectory and head direction correlates during spatial behaviors (Ito et al., 2015; Jankowski et al., 2014, 2015; O'Mara and Aggleton, 2019). At the network level, inactivation of RE reduces HC-PFC oscillatory synchrony across theta, beta, and gamma bands during wakefulness (Kafetzopoulos et al., 2018; Totty et al., 2023), as well as slow oscillations and gamma during sleep (Ferraris et al., 2018; Hauer et al., 2019). Notably, RE lesions specifically disrupt tasks requiring HC-PFC coordination but leave HC- or PFC-dependent tasks largely intact (Hembrook and Mair, 2011; Hembrook et al., 2012; Dolleman-van der Weel et al., 2009; Loureiro et al., 2012; Ito et al., 2015; Cassel et al., 2013). Recent evidence from sleep recordings in cats further indicates that reuniens activity is strongly modulated by hippocampal oscillations and precedes the onset of cortical spindles (Basha et al., 2025). Based on existing literature, RE has been suggested as an important node in the extended network underlying spatial cognition (Griffin, 2021).

Despite accumulating evidence for functional interactions between RE, HC, and PFC, it remains unclear whether RE acts as a passive relay that forwards signals between HC and PFC, or as an active integrator that transforms information within this network. If RE functions as a passive relay, its activity should closely mirror its inputs, with learning-related changes propagating symmetrically across HC-RE and RE-PFC pathways. In contrast, an active integrator role would be reflected in more complex dynamics, such as asymmetric directional coupling, pathway-selective modulation, or behavioral state-dependent interactions. Clarifying the role of RE is crucial for understanding the circuit mechanisms underlying spatial cognition.

Neural oscillations offer valuable insight into the mechanisms of inter-regional communication. Synchronous rhythmic activity, as detected in local field potentials (LFPs), provides a temporal framework for coordinating neuronal firing across brain regions, thereby facilitating the encoding, transfer, and retrieval of information (Fries, 2005; Engel and Fries, 2010; Colgin, 2016). In rodents, hippocampal theta oscillations (6–11 Hz) are considered essential for HC-PFC communication. Multiple studies have reported phase-locking of prefrontal neurons to hippocampal theta in animals performing spatial working memory tasks, with the strength of coupling correlating with performance (Jones and Wilson, 2005; Benchenane et al., 2010; Hyman et al., 2010; Hallock et al., 2016; de Mooij-van Malsen et al., 2023). Granger causality has been used alongside coherence to demonstrate bidirectional HC-PFC theta interactions during a memory task (Place et al., 2016), and spatial task performance is associated with increased HC-PFC coherence in gamma bands (Spellman et al., 2015; Bygrave et al., 2019; de Mooij-van Malsen et al., 2023). However, it remains largely unknown whether these oscillatory interactions are mediated through RE or arise independently of it.

Here, we reanalyzed data from a previous study (Baumann et al., 2026) to investigate functional interactions within the HC-RE-PFC network as rats learned to navigate a complex maze across multiple days. Using measures of coherence and Granger causality, we characterized how local oscillatory activity, long-range functional coupling, and directional connectivity evolved with learning. We hypothesized that spatial learning would be accompanied by dynamic changes in the HC-RE-PFC network, including alterations in the directionality of information flow. Our results reveal a striking asymmetry: learning selectively increased HC-to-RE information flow, particularly in the beta (15–30 Hz) band, while RE-outgoing interactions with HC and PFC remained stable or decreased. These findings are inconsistent with a simple relay account and instead support a model in which RE actively participates in spatial information processing by receiving and integrating hippocampal input.

## Results

To monitor the dynamics of neural activity and cross-regional interactions during spatial navigation, adult male rats were chronically implanted with electrodes in the deep layers of the prelimbic cortex (PrL, medial PFC), CA1 of the dorsal hippocampus (HC), and the midline thalamic nucleus reuniens (RE; Figure 1a). PFC and HC electrode positioning was guided electrophysiologically during implantation, while RE placement was subsequently verified histologically in each rat (Supplementary Figure S1). Local field potentials (LFPs, 1–500 Hz) were recorded simultaneously from all regions throughout the experiment.

**Figure 1:**
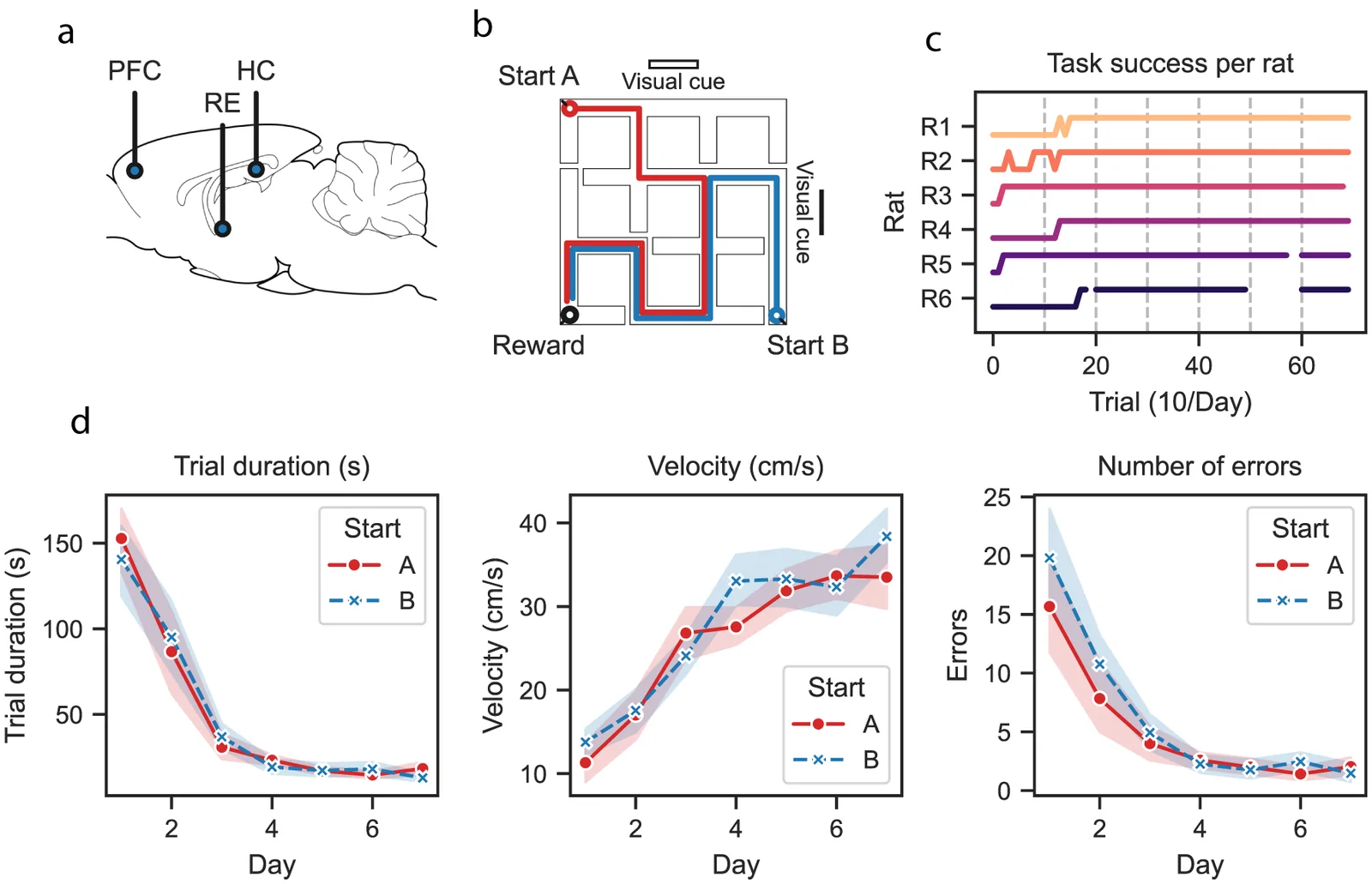
Experimental setup and behavioral dynamics during spatial learning. **(a)** Schematic electrode placement in the rat brain, targeting medial PFC, RE, and dorsal HC. Figure based on Paxinos and Watson (2006). **(b)** Maze layout. The colored lines indicate the shortest routes from the starting points to the reward. Two distinct visual cues were affixed to the curtain surrounding the maze to serve as allocentric reference points. **(c)** Task success per rat. An increase in the line indicates a successful trial, i.e., the rat reached the reward within 3 min. All rats discovered the reward location within the first 2 days. Gaps indicate missing trials due to tracking failure. **(d)** Behavioral metrics across days, separated by start. Shading indicates 95% confidence intervals. Behavior did not differ notably between starting points.

For spatial learning, an elevated maze with a crossword-like layout containing multiple intersections and dead ends was used; there were two starting points (A and B) and one reward port (Figure 1b). Two salient visual cues provided an allocentric reference frame. Animals were habituated to the environment, but the maze layout and reward location were novel at the beginning of learning. On each trial, rats navigated from one of the two starting points to a fixed reward location. Both starting points were equidistant from the goal. The optimal path required 6 and 4 navigational decisions (maze intersections) for start A and B, respectively. Rats performed 10 trials per session over 7 days; to minimize the involvement of procedural memory, training was stopped after seven sessions, which appeared sufficient to achieve asymptotic performance.

All rats located the reward for the first time within 1–2 sessions (3–19 trials). After initial discovery, 4/6 rats consistently located the reward on all subsequent trials within a 180-s cutoff time, while the remaining two animals required additional trials to stabilize performance (Figure 1c). Representative trajectories across learning are shown in Supplementary Figure S2. Trials before reward discovery were characterized by variable behavior, including pauses, grooming, and prolonged periods of immobility, while trials after reward discovery became progressively more reward-oriented. We quantified behavioral performance by trial duration, average velocity, and the number of navigation errors (Figure 1d). For incomplete trials in which no reward was obtained, a cutoff time of 180 s was assigned as the trial duration. Errors at intersections were defined as choices that did not reduce distance to the goal. Across days, performance improved significantly (two-way repeated-measures ANOVA, day × start, FDR-corrected; Rat 6 was omitted from this analysis due to missing behavioral data on day 6), with strong main effects for all behavioral metrics (all $F_{\left(6,24\right)}\geq 11.85,p<0.001,\eta_{G}^{2}\geq 0.64$). Overall, rat behavior did not differ on A- and B-trials; there was a small main effect of start for velocity ($F_{\left(1,4\right)}=11.24,p=0.047,\eta_{G}^{2}=0.01$), due to a somewhat higher velocity during trials starting from B on days 4 and 7 (Figure 1d), as well as significant interactions for trial duration and velocity ($F_{\left(6,24\right)}\geq 3.71,p\leq 0.018,\eta_{G}^{2}\geq 0.03$); however, *post-hoc* pairwise comparisons were not significant (all days *p*≥0.092, *post-hoc* pairwise *t*-tests, FDR-corrected). Since these effects were not consistent, A- and B-trials were pooled for subsequent electrophysiological analyses.

###  Spatial learning drives region-specific changes in LFP spectral power

To explore learning-associated local oscillatory changes, we analyzed region-specific LFP spectral power across 7 days of learning. Based on prior literature, we selected four frequency bands: theta (6–11 Hz), beta (15–30 Hz), low gamma (30–60 Hz), and high gamma (60–120 Hz). Power spectral density (PSD) was computed using Welch's method for a total of 364 artifact-free trials (*n* = 6 rats, 32–70 trials per rat). Results are summarized in Figure 2. All brain regions exhibited prominent theta peaks around 7.5–8 Hz (Figure 2a). Since it is well-established that theta power in HC is velocity-modulated (Whishaw and Vanderwolf, 1973; Ahmed and Mehta, 2012; Kennedy et al., 2022), and the average running speed progressively increased with better task performance (Figure 1d), we estimated the effect of learning using linear mixed-effects modeling with trial-averaged velocity as a covariate (see Methods for details), separately for each brain region:

theta power ~ C(day) + velocity + (1|rat).

**Figure 2:**
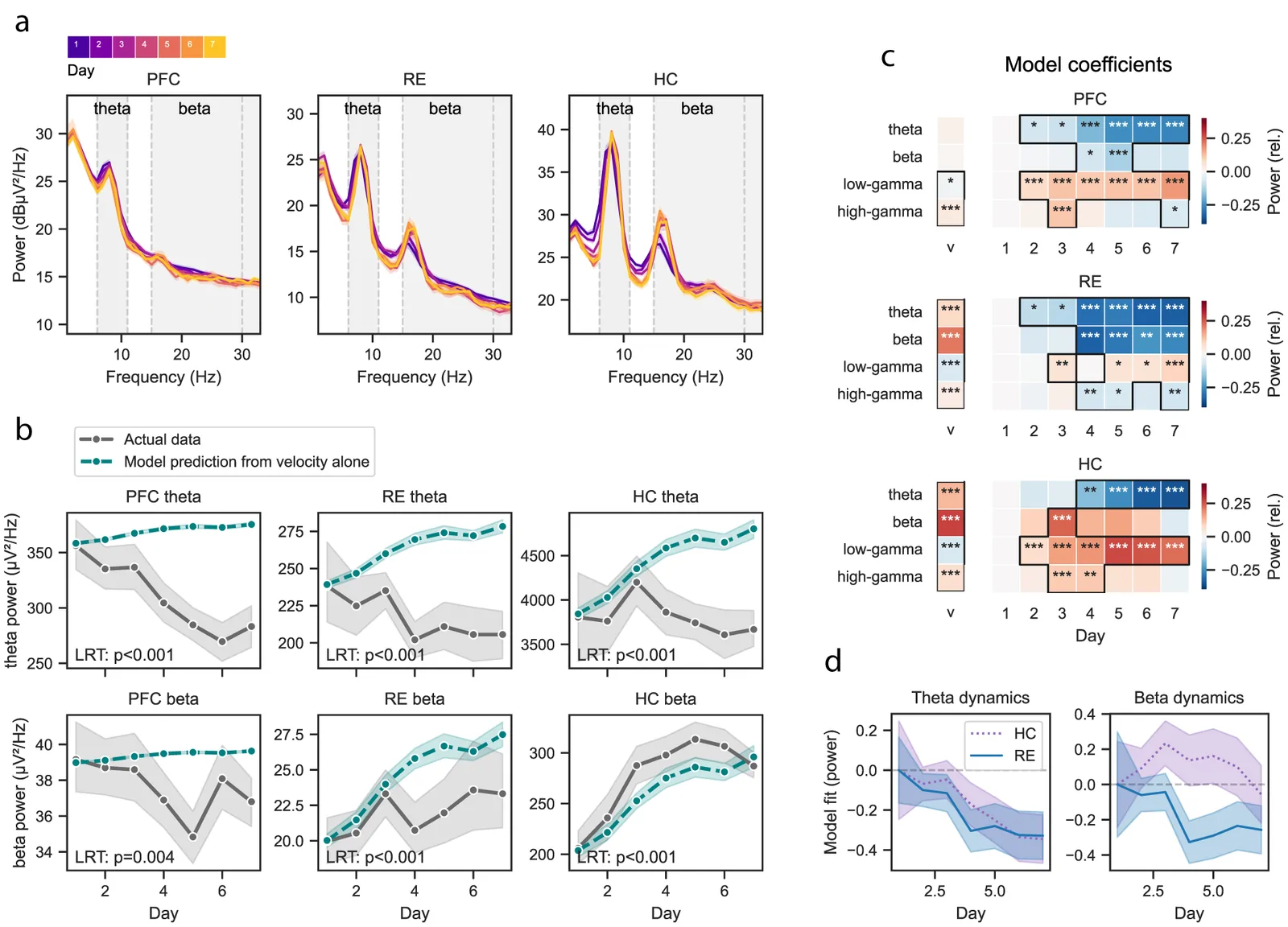
**(a)** Power spectral density across days (color gradient). All regions show a clear theta peak at ~8 Hz, as well as a second, lower, beta peak at ~16 Hz, which appears to be the second theta harmonic. Band power was estimated by averaging power across predefined ranges (gray bars); the plot shows log-transformed power for visibility, but untransformed values were used for model estimates. For visibility, spectra in the main panel are restricted to frequencies containing the prominent theta and beta peaks; spectra including the gamma ranges used for statistical analyses are shown in Supplementary Figure S3. **(b)** Effects of velocity. Linear mixed-effects model predictions for band power (top: theta, bottom: beta) based on the estimated velocity coefficient (and intercept) only. Shading indicates 95% confidence intervals. Likelihood-ratio Test (LRT) labels indicate whether adding day significantly improved model fit compared with the reduced velocity-only model (LRT, fdr-corrected). **(c)** Effects of learning. Linear mixed-effects model coefficients for velocity (v) and each day, normalized to the intercept (day 1). Velocity coefficients are shown for 10 cm/s increments to improve visibility. Stars indicate significant increases or decreases (*z*-test against 0, fdr-corrected). **(d)** Model fits revealed dissociable dynamics between HC and RE theta and beta when controlling for velocity. Normalized model coefficients per day (as in **c**). Shading indicates 95% confidence intervals.

The effect of learning, C(day), was treated as a categorical variable to avoid the assumption of monotonicity. Consistent with previous findings, velocity significantly predicted theta power in HC (β = 42.23, *SE* = 6.50, *z* = 6.49, *p* < 0.001, *z*-test, fdr-corrected). RE theta was also significantly modulated by velocity (β = 1.72, *SE* = 0.42, *z* = 4.10, *p* < 0.001), but not PFC theta (β = 0.75, *SE* = 0.48, *z* = 1.57, *p* = 0.14, Figures 2b, c).

To test if there was an additional effect of learning, i.e., velocity-independent theta power variability across days, each model was compared to a reduced model without day:

theta power ~ velocity + (1|rat).

Including day as a categorical variable significantly improved the model fit across all brain regions (all $\chi_{\left(6\right)}^{2}\geq 44.40,p<0.001$, likelihood-ratio test, fdr-corrected), indicating learning-related changes in theta power that could not be explained by differences in velocity alone. In all regions, the model coefficients for day showed a significant decrease in theta power across learning (e.g., Day 7: all *z* ≤ −5.40, *p* < 0.001, Figure 2c). That is, HC and RE theta power were lower than expected based solely on the positive relationship between velocity and theta power, while PFC theta power decreased across learning despite no significant velocity dependence.

In addition to theta, RE and HC showed a pronounced peak in the beta band (16–16.5 Hz, Figure 2a). We repeated the model fits for the HC and RE beta power and found a significant positive relationship with the velocity (HC beta: β = 4.07, *SE* = 0.40, *z* = 10.26, *p* < 0.001, RE beta: β = 0.33, *SE* = 0.03, *z* = 9, 48, *p* < 0.001, Figure 2b). Including day improved model fits for both regions ($\chi_{\left(6\right)}^{2}\geq 35.05,p<0.001$), again indicating learning-related modulation beyond velocity.

The width (13–19 Hz) and peak frequency (16 Hz) of the beta band peak (Figure 2a) suggest that it may have been caused by a theta harmonic (a signal at an integer multiple frequency of the original signal, caused by Fourier decomposition) rather than an independent rhythm. Consistent with this interpretation, when controlling for velocity, RE beta showed similar dynamics in theta and beta, with a significant decrease in RE beta power from days 4 to 7 (e.g., Day 4: β = −5.11, *SE* = 0.96, *z* = −5.32, *p* < 0.001). In HC, however, beta exhibited a transient increase early in learning (Day 3: β = 34.82, *SE* = 9.67, *z* = 3.60, *p* = 0 < 0.001), dissociating it from theta dynamics (Figure 2d). Therefore, if harmonic, HC and RE beta must have different non-linear relationships with theta, suggesting different computational or cognitive functions. In this sense, the results support a learning-related circuit reorganization during spatial learning, even though the observed beta might have been a Fourier-based byproduct of theta.

Finally, low and high gamma power were also modulated by velocity and learning in all brain regions (velocity: all |*z*|≥2.46, *p* = 0.025, model comparison: all $\chi_{\left(6\right)}^{2}\geq 31.14,p<0.001$). Specifically, increased HC and PFC low-gamma power accompanied efficient task performance (e.g., Day 7: *z*≥7.56, *p* < 0.001); whereas a significant high-gamma power increase was observed earlier in learning (e.g., Day 3: *z*≥3.71, *p* ≤ 0.001 Figure 2c). In RE, we detected transient increases in low-gamma power and decreases in high-gamma power, but the effects were inconsistent and relatively weak (e.g., Day 7: |*z*|≥2.78, *p* ≤ 0.01). For a full list of model coefficients, see Supplementary Tables S1–S4.

Overall, our analyses of the band-limited LFP power across learning revealed region-specific spectral changes beyond those attributable to gradual improvement of procedural aspects of task performance, such as faster running. HC, RE, and PFC exhibited dissociable, band-specific temporal dynamics indicative of differential engagement in local computation and circuit reorganization. The findings suggest that spatial learning altered neural dynamics and local information processing independently of accompanying changes in locomotor behavior.

###  Beta coherence reveals learning-associated network reorganization

Based on the observed LFP band-limited power dynamics, we hypothesized that spectral changes may have been accompanied by corresponding increases and decreases in cross-regional functional connectivity within the HC-RE-PFC network. To test this hypothesis, we estimated spectral coherence between each pair of brain regions (see Methods). All pairs exhibited strong theta coherence, with an additional beta peak in HC-RE and HC-PFC (Figure 3a).

**Figure 3:**
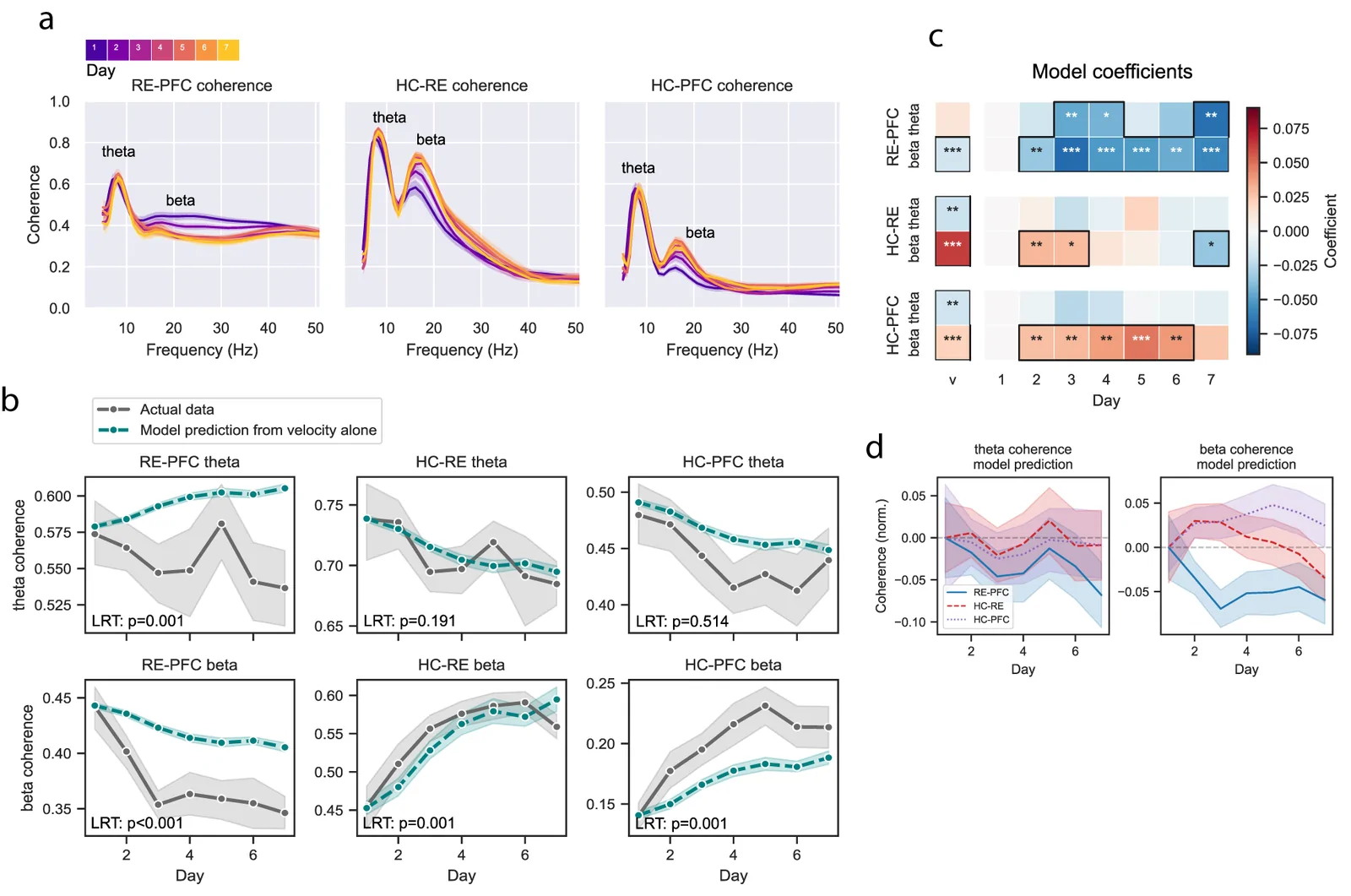
Coherence as a measure of functional connectivity between regions. **(a)** Spectral coherence across days (color gradient). **(b)** Effects of velocity. Linear mixed-effects model predictions for coherence (**top**: theta, **bottom**: beta) based on the estimated velocity coefficient (and intercept) only. Shading indicates 95% confidence intervals. Likelihood-ratio test (LRT) labels indicate whether adding day significantly improved model fit compared with the reduced velocity-only model (LRT, fdr-corrected). **(c)** Linear mixed-effects model coefficients for velocity (v) and each day, relative to the intercept (day 1). Velocity coefficients are shown for 10 cm/s increments to improve visibility. Stars indicate significant increases or decreases (*z*-test against 0, fdr-corrected). **(d)** Effects of learning. Model predictions for coherence across days (i.e., without the effect of velocity). While generally high, theta coherence showed little variation across days (or velocity), while beta coherence varied substantially across pairs.

To estimate the effect of learning on cross-regional coherence while accounting for velocity modulation, we fitted linear mixed-effects models (as above) to band-averaged theta and beta coherence for each region pair. Likelihood-ratio tests (LRTs) comparing the full models with reduced models containing velocity alone showed a significant effect of day for RE-PFC theta coherence and for RE-PFC beta coherence in all three region pairs (all $\chi_{\left(6\right)}^{2}\geq 22.76,p\leq 0.001$), but not for HC-RE or HC-PFC theta coherence ($\chi_{\left(6\right)}^{2}\leq 9.26,p\geq 0.191$; Supplementary Table S5). In the following, we present the most striking results; for a full list of model coefficients, see Supplementary Tables S5–S8. Cross-regional theta coherence showed the highest magnitude across all region pairs but, consistent with the model comparisons, exhibited limited learning-related modulation. HC-RE and HC-PFC theta coherence slightly decreased with velocity (HC-RE: β = −0.002, *SE* = 0.001, *z* = −2.87, *p* = 0.012, HC-PFC: β = −0.002, *SE* = 0.001, *z* = −2.89, *p* = 0.010); RE-PFC theta coherence showed no velocity modulation but significant decreases for some days (Days 3,4,7: β ≤ −0.042, *SE*≥0.015, *z* ≤ −2.42, *p* ≤ 0.033; Figures 3b, c).

Compared to theta, beta coherence showed more variable dynamics (Figures 3b–d). All region pairs showed significant velocity and learning-related modulation of beta coherence. Notably, beta synchrony between HC and the other two regions increased both with velocity (HC-RE: β = 0.006, *SE* < 0.001, *z* = 14.13, *p* < 0.001; HC-PFC: β = 0.002, *SE* < 0.001, *z* = 5.28, *p* < 0.001), and across days (e.g., HC-RE Day 2: β = 0.030, *SE* = 0.010, *z* = 3.111, *p* = 0.007; HC-PFC Day 5: β = 0.048, *SE* = 0.012, *z* = 4.041, *p* < 0.001). At the same time, beta synchrony between RE and PFC decreased, with significant model coefficients for both velocity (β = −0.002, *SE* < 0.001, *z* = −3.73, *p* = 0.001) and learning across all days (β ≤ −0.034, *SE* ≤ 0.014, *z* ≤ −3.20, *p* ≤ 0.005).

Spurious coupling between regions may arise from field spread across tissue (volume conduction), which is particularly prominent at lower frequencies (Bastos and Schoffelen, 2016). To test whether coherence was caused by volume conduction, we also computed the volume-conduction-resistant weighted phase-lag index (WPLI) (Vinck et al., 2011). The WPLI showed large non-zero theta and beta peaks (Supplementary Figure S4), suggesting genuine interactions.

Overall, the analysis of cross-regional coherence revealed learning-dependent changes in functional coupling that were, at least in part, dissociable from effects of locomotion. In the beta frequency range in particular, we observed distinct temporal profiles across all region pairs, which may reflect functional network reorganization associated with spatial learning.

###  Spatial learning is accompanied by an increase in HC-to-RE information flow

To further explore neural network dynamics associated with spatial learning, we next assessed the directionality of cross-regional interactions using Granger causality (GC). Consistent with the spectral analyses described above, frequency-resolved GC showed prominent theta and beta peaks across all region pairs, with the strongest effects observed for HC-to-RE GC (Figure 4a). Notably, GC peak frequencies in the theta and beta bands were shifted away from the harmonic peaks observed for power spectral density (see Results above), with lower theta (6.5–7 Hz) and higher beta (17–21.5 Hz).

**Figure 4:**
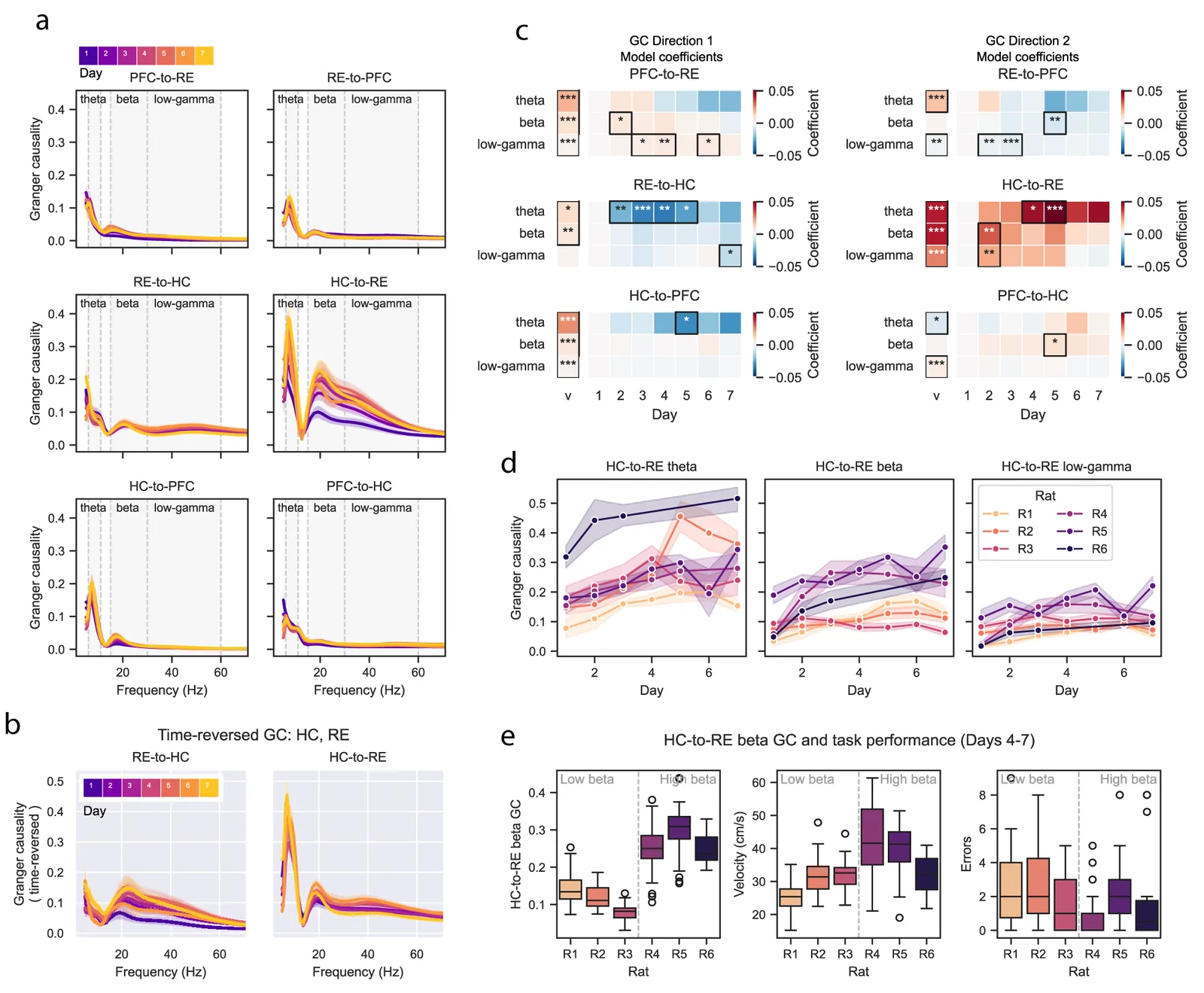
**(a)** Granger causality (GC) across days (color gradient). Shading indicates 95% CI. Directions are shown side-by-side with fixed scaling for comparison. HC-to-RE theta, beta, and low-gamma GC were much higher than any other result, and show a clear trend across days. **(b)** Time-reversed GC for the main HC-to-RE result. Compared to (forward) GC in **(a)**, the patterns are clearly reversed, suggesting a genuine effect. However, the GC magnitude, particularly for HC-to-RE theta, did not reverse, suggesting that the magnitude of the effect may have been caused by large differences in HC theta power compared to RE. **(c)** Model coefficients. Stars indicate significant differences from day 1 (linear mixed-effects models, fdr-corrected). HC-RE GC was much higher in absolute magnitude, but other region pairs also had significant GC dynamics (see text). **(d)** Band-averaged HC-to-RE GC across days, split by rat. GC was broadly distributed across rats, with some showing a much stronger increase across learning than others. For beta, rats appeared to split into two groups during learning (low and high beta; see **(e)**). **(e)** Low and high HC-to-RE beta GC groups (days 4–7, see **(d)**), compared to task performance. While the “high beta” group had significantly higher HC-to-RE beta GC (*t*_(4)_ = −6.11, *p* = 0.004, independent *t*-test), this difference did not directly translate to behavior (velocity: *t*_(4)_−2.20, *p* = 0.09, errors: *t*_(4)_ = 1.43, *p* = 0.23).

For each region pair and direction of interaction, we fitted linear mixed-effects models to band-averaged theta, beta, and low-gamma GC. The low-gamma range was included due to a visible inflection in HC-to-RE low-gamma GC (Figure 4a). Mixed-effects modeling revealed several significant changes in the directionality of cross-regional interactions both across learning and attributable to velocity (Figure 4c). For model comparisons and a full list of model coefficients, see Supplementary Tables S9–S15. The most striking effects were observed for the HC-RE pair, which showed substantial changes in information flow across learning. HC-to-RE GC was both positively correlated with velocity (all bands, *z*≥6.45, *p* < 0.001, *z*-test, fdr-corrected) and showed early to intermediate increases with learning (e.g., GC-beta and low-gamma, day 2: *z*≥3.31, *p* ≤ 0.005, GC-theta, days 4-5: *z*≥2.48, *p* ≤ 0.049). LRTs confirmed significant effects of day on HC-to-RE GC in theta, beta, and low-gamma bands (all $\chi_{\left(6\right)}^{2}\geq 20.55,p\leq 0.004$; Supplementary Table S9). In the opposite direction, RE-to-HC theta significantly decreased after day 1 (GC-theta, days 2-5: *z* ≤ −2.72, *p* ≤ 0.027).

Notably, HC-to-RE GC showed substantial inter-individual variability, with HC-to-RE beta GC in particular appearing to converge into two groups (low- and high-beta, respectively, Figures 4d, e). Given the strong model fit to velocity, we tested whether rats with higher HC-to-RE beta GC also performed better on the task. A direct comparison between groups confirmed a significantly higher HC-to-RE beta GC for the high-beta rats (R4-6) compared to the low-beta group (R1-3, *t*_(4)_ = 4.99, *p* = 0.001, independent *t*-test, fdr-corrected), but they did not run significantly faster (*t*_(4)_ = −2.20, *p* = 0.186) nor made fewer errors (*t*_(4)_ = 1.43, *p* = 0.199, Figure 4e). Although preliminary, these observations suggest that HC-RE interactions may be highly variable across animals despite similar performance.

The other region pairs showed less pronounced changes in the directional interaction. The results suggest a top-down prefrontal influence on RE, with a significant learning-related GC increase for PFC-to-RE low-gamma (days 3,4,6: *z*≥2.70, *p* ≤ 0.028) and decrease in the opposite direction (RE-to-PFC low-gamma, days 2–3: *z* ≤ −3.15, *p* ≤ 0.008). The HC-PFC pair showed almost no changes across learning; HC-to-PFC theta was strongly modulated by velocity (β = 0.10, *SE* = 0.03, *z* = 4.06, *p* < 0.001) and generally higher than theta in the opposite direction, suggesting HC-to-PFC theta information flow primarily, which appeared to be unrelated to learning.

The estimation of GC is affected by the relative oscillatory power of the respective brain regions, particularly for volume conduction, which may give rise to spurious GC (Stauch et al., 2021). This effect can be tested by estimating GC for time-reversed signals, which leaves the relative oscillatory power unchanged but reverses directionality (Haufe et al., 2012; Vinck et al., 2015). To confirm our findings, we therefore estimated time-reversed GC across all region pairs (Supplementary Figure S5), but in particular for HC-to-RE GC (Figure 4b). Our (forward) GC results include two aspects: a magnitude that indicates the direction of information flow, and a change over days that suggests changes in cross-regional communication over time. Time-reversed HC-to-RE (theta) GC did not reverse in *magnitude*, suggesting that the relative directionality may primarily reflect a generally higher HC theta power compared to RE. However, the *change over days* did reverse, confirming that task learning led to an increase in HC-to-RE information flow. The directionality of PFC-RE GC interactions also appeared to reverse for time-reversed GC, although the effects were less pronounced due to a generally lower GC magnitude (Supplementary Figure S5).

Altogether, GC revealed that information flow during spatial learning is reorganized in a frequency- and direction-specific manner, rather than showing a uniform increase or decrease in functional connectivity across the brain. The findings indicate that, during spatial navigation, information predominantly flows from the HC to other brain regions, with transient top-down influences from PFC to RE observed during the early stages of learning. Taken together with results from LFP spectral power and coherence, the most robust task- and learning-related alterations appear to occur between HC and RE in the 15–30 Hz beta band.

## Discussion

In the present study, we characterized dynamic changes in local and long-range functional connectivity within the HC-RE-PFC network as rats learned to navigate a complex maze to obtain a reward. Learning-related changes were most prominent in cross-regional directional measures of information flow, as revealed by striking increases in HC-to-RE theta, beta, and low-gamma Granger causality. Our results confirm the involvement of HC-PFC interactions in spatial cognition, and additionally provide new evidence that learning-related reorganization within the HC-RE-PFC network is primarily driven by changes in HC-to-RE communication. Early and intermediate phases of learning were associated with increases in HC-to-RE theta and beta Granger causality (GC), as well as increased PFC-to-RE low-gamma GC. In contrast, several RE-outgoing interactions decreased, including RE-to-PFC gamma GC and RE-to-HC theta GC. Taken together, our findings suggest that, in addition to learning-related modulation of the strength of cross-regional communication, the transition to more efficient navigation may require rerouting of information flow. The observed changes in local activity and long-range communication identify the RE as a potential integrator of hippocampal input rather than passively relaying it to PFC.

###  HC-PFC interactions support spatial behavior

Our findings align with extensive literature on the importance of HC-PFC interactions for spatial cognition (e.g., Ito, 2018; Eichenbaum, 2017; Buzsáki, 1996; Colgin, 2011; Wirt and Hyman, 2017), where theta synchrony between HC and PFC in particular has long been implicated in spatial working memory and decision-making (Jones and Wilson, 2005; Hyman et al., 2010; Colgin, 2011; de Mooij-van Malsen et al., 2023). Surprisingly, however, we found that HC-PFC theta coherence was already elevated at the onset of learning, when the rats exhibited predominantly exploratory behavior; its strength was neither affected by a switch to goal-directed navigation nor did it change with more efficient task performance. In contrast, HC-RE interactions showed pronounced learning-related modulation, suggesting that the primary locus of network reorganization may lie within the HC-RE pathway rather than direct HC-PFC interactions.

The absence of robust learning-related theta modulation does not imply that theta is unimportant for HC-PFC communication. Rather, the findings suggest that theta synchrony may provide a stable communication scaffold upon which more dynamic beta- and gamma-range interactions are organized. Under this view, theta reflects the persistent anatomical and functional coupling of the HC-RE-PFC network, whereas beta and gamma interactions may encode task-specific demands and learning processes. However, it is also possible that the trial-averaged analyses obscured transient theta dynamics associated with specific behavioral events. Future analyses aligned to maze intersections, reward acquisition, or error correction may reveal event-specific theta dynamics that were not detectable at the temporal resolution used here.

###  RE as an active receiver of hippocampal input

The most prominent learning-related effect was a striking increase in HC-to-RE Granger causality across the beta and low-gamma bands, accompanied by weaker or absent changes in RE-outgoing connections. This directional asymmetry is inconsistent with a purely relay-like role of RE, in which learning-related changes would be expected to propagate similarly across both HC-RE and RE-PFC interactions. These observations suggest that RE may transform, gate, or redistribute hippocampal input rather than merely forwarding it to the PFC.

One possibility is that the observed HC-RE beta coupling helps maintain task-relevant information during navigation. Beta oscillations have been proposed to play a broader role in maintaining task-relevant representations during working memory and decision-making (Lundqvist et al., 2016; Schmidt et al., 2019). Our findings may be consistent with this framework: HC-to-RE beta GC increased during the early stages of task acquisition, when behavioral performance was also changing most strongly. The temporal correspondence is consistent with a role in establishing task-relevant representations or action policies, although the absence of a direct relationship with error rate prevents us from assigning the effect specifically to successful spatial learning.

This aligns with recent work in which HC-RE beta synchrony predicted performance in a sequence memory task and was proposed to reflect a neurobiological memory buffer (Jayachandran et al., 2023). Although the present data cannot directly test this hypothesis, increased HC-to-RE beta communication during early spatial learning is consistent with a similar mechanism.

An alternative interpretation is that the increase in HC-RE beta reflects the acquisition of procedural action sequences. However, lesion evidence argues against a purely procedural account: RE is required for tasks that depend on HC-PFC coordination (Davoodi et al., 2009; Mei et al., 2018; Hembrook and Mair, 2011; Jayachandran et al., 2019), but not for tasks depending on HC (Dolleman-van der Weel et al., 2009; Hembrook et al., 2012; Loureiro et al., 2012; Cassel et al., 2013) or PFC alone (Ito et al., 2015). Moreover, post-trial RE lesions do not impair memory consolidation (Mei et al., 2018).

A second notable finding was the shift in PFC-RE interactions across learning: PFC-RE beta coherence was highest during initial maze exposure and declined with learning, while PFC-to-RE low-gamma GC increased. This may reflect a shift from early novelty- or exploration-related prefrontal engagement toward more hippocampus-driven interactions as spatial representations consolidated. Although speculative, this interpretation is consistent with recent evidence for a specific role of PFC-RE communication in non-HC-dependent memory (Ciacciarelli et al., 2025). Given RE's extensive anatomical connectivity, for example, to the anterior cingulate, retrosplenial, perirhinal, and entorhinal cortices, as well as the claustrum (Mathiasen et al., 2020), it is also likely that learning-related hippocampal input was redistributed to targets beyond the ones recorded here, further supporting a role for RE as an active network component rather than a point-to-point relay.

###  Beta oscillations: Theta harmonic or distinct network rhythm?

A central issue in interpreting the results concerns the harmonic nature of the observed beta-band oscillations. A fundamental limitation of Fourier-based signal analysis is the assumption of sinusoidal components; the spectral decomposition of non-sinusoidal signals will therefore lead to the appearance of harmonic peaks at integer multiples of the fundamental frequency. Such waveform-dependent harmonics are common in electrophysiological recordings and reflect underlying neuronal and circuit properties rather than independent oscillatory generators (Cole and Voytek, 2017; Gao et al., 2017).

In the present dataset, the narrow ~17 Hz peak observed in HC and RE power spectra closely matches the second harmonic of the dominant ~8.5 Hz theta rhythm. Indeed, such harmonics have previously been attributed to changes in running speed (Sheremet et al., 2016). Consequently, some of the observed beta-range power and connectivity likely reflect learning-related changes in theta waveform asymmetry associated with locomotion rather than independent beta dynamics.

Several observations, however, argue against a purely harmonic interpretation. HC-to-RE beta and low-gamma GC showed non-harmonic peak frequencies and much wider spectral profiles than expected from a harmonic account, even when considering higher-order harmonics. Moreover, theta- and beta-range measures displayed distinct learning-related dynamics, suggesting at least partial functional dissociation. Finally, time-reversed GC analyses supported the specificity of beta- and low-gamma-range interactions, whereas theta and its harmonic components showed much weaker reversals (Supplementary Figure S5a). We therefore interpret the beta-range findings as reflecting a mixture of theta harmonics and distinct network interactions.

Importantly, these possibilities are not mutually exclusive. Even purely harmonic components remain physiologically meaningful as they reflect underlying changes in neural waveform shape and circuit dynamics and should not be dismissed as analytical artifacts (Cole and Voytek, 2017; Lozano-Soldevilla, 2018). Thus, regardless of whether the observed beta activity represents an independent oscillation or a theta harmonic, the convergence of coherence, GC, and time-reversed GC analyses supports a learning-related strengthening of HC-to-RE interactions.

###  Study limitations and future directions

Several limitations should be acknowledged. First, disentangling velocity-related from learning-related effects is inherently difficult, as running speed increased alongside task performance. Including velocity as a covariate in the mixed-effects models only partially addresses this confound, as non-linear or state-dependent locomotor effects remain. Nevertheless, the early rise in HC-to-RE beta and low-gamma GC indicates that the neural signature increased before velocity, suggesting that these neural changes may not simply reflect improved locomotion.

Second, the LFP-based connectivity measures constrain the network-level role of RE, yet cannot distinguish genuine cross-regional interactions, for example, common inputs originating from a third region. Another methodological approach is required to reveal the representational content of the underlying network activity, for example, pathway-specific manipulations establishing causal relationships between HC-RE communication and behavior. Therefore, the present results do not distinguish whether RE neurons encode or mediate spatial, mnemonic, action-related, or other task-relevant information, nor does it address the cellular mechanisms underlying the observed interactions.

Finally, the trial-averaged analyses may have obscured rapid, event-locked dynamics related to ongoing behavior; these and other important questions are beyond of the scope of the present study and shall be addressed in future studies.

## Materials and methods

###  Animals

The data were obtained from six adult male rats (Sprague-Dawley, Charles River Laboratories) weighing 300–350 g at the start of the experiment. Animals were housed under controlled conditions (20–30°C, 40%–60% humidity, 12h light/dark cycle). All experiments were performed during the dark phase. Prior to surgery, rats were group-housed with *ad libitum* access to food and water. After implantation, animals were single-housed to prevent damage to the implant. During behavioral testing, rats were food-restricted to 90% of *ad libitum* body weight to ensure appetitive motivation.

All procedures complied with Directive 2010/63/EU and the German Animal Welfare Act (TierSchG, TierSchVersV). Protocols were approved by the local ethics committee (§15 TierSchG) and the state authority (Regierungspräsidium Tübingen, Baden-Württemberg, Referat 35, Veterinärwesen, permit KY01/15).

###  Surgical procedures

Surgeries were performed under standard aseptic conditions and described in detail elsewhere (Mei et al., 2018). Animals were anesthetized with isoflurane (5% induction, 1.5%–2% maintenance) and head-fixed in a stereotaxic frame (David Kopf Instruments, Tujunga, CA, USA) with head angle adjusted to 0°. Body temperature (~37 °C), heart rate, and oxygen saturation were continuously monitored.

Following local anesthesia (lidocaine), the skull was exposed, and craniotomies were made above right-hemisphere targets according to the rat brain atlas (Paxinos and Watson, 2006): PFC (redPrL, AP/ML = 3.1/0.8 mm), HC (CA1, AP/ML = −3.8/2.4 mm), and RE (AP/ML = −1.8/1.3 mm). Single-tipped platinum-iridium electrodes (FHC Inc. Bowdoin, ME, USA) were chronically implanted at PFC (DV = 3.4 mm), HC (DV = 2 mm), and RE (DV = 6.8 mm, 10° medial-lateral angle to avoid the sagittal sinus). PFC and HC electrodes were implanted under electrophysiological control to ensure positioning in PrL and CA1, respectively.

Five stainless steel screws (0.86 mm diameter) were fixed to the skull and served as ground/reference for the implant. A 3D-printed plastic frame and copper mesh provided electrical shielding and were secured to the skull with tissue adhesive. The entire assembly was fixed with dental cement (RelyX Unicem 2 Automix, 3M).

During postoperative recovery (5–7 days), rats received antibiotics (Baytril 5 mg/kg i.p., Bayer AG, Leverkusen, Germany) and analgesics (Rymadil 2.5 mg/kg i.p., Zoetis, Parsippany, New Jersey, USA).

###  Behavioral apparatus and training

Behavioral testing took place in a custom-built elevated maze (130 × 130 cm, black PVC) consisting of a 4 × 4 lattice of arms (10 cm wide, 2 cm rims), elevated 80 cm above the floor. A reward port delivered chocolate milk (0.6 ml) upon nose poke. Allocentric visual cues were provided by two large posters placed on the surrounding curtains. Experiments were conducted under dim light.

Animals were habituated (1–3 sessions) on a simplified linear track configuration until they reliably approached the feeder and consumed rewards without signs of anxiety.

During the task, nine barriers (30 cm high, 25–40 cm wide) created a fixed maze configuration with multiple decision points. Rats were released at one of two pseudorandomized starting positions and had 180 s to reach the reward location. Trials ended upon reward consumption or timeout. For incomplete trials (i.e., trials in which no reward was obtained), a timeout of 180 s was assigned as trial duration. Trial start and end timestamps were recorded by a light barrier and the nose port, respectively. Between trials, rats rested in a waiting box for 3–5 min while the maze was cleaned to minimize olfactory cues. Each rat completed 10 trials per day over 7 days. Trials with tracking failure (14 trials, 3.3%, Figure 1c) were excluded.

The complex maze, multiple starting locations, and allocentric cues were intended to promote HC-dependent navigation and spatial memory compared to simpler habitual strategies (Mei et al., 2018). Specifically, the current design minimized procedural learning by requiring navigation along two different trajectories through multiple starting locations, a complex maze configuration with multiple choice points, and avoiding overtraining. In addition, a previous study with the same maze (Mei et al., 2018) found that animals used the allocentric visual cues, indicating a spatial strategy.

###  Behavioral tracking

Movement was recorded at 25 Hz using an overhead camera that tracked two head-mounted LEDs synchronized with the electrophysiological recording system. Head position and orientation were estimated from LED coordinates, and missing samples were linearly interpolated. Velocity was derived from position and smoothed using a Savitzky-Golay filter. Body posture was not tracked.

Behavioral performance was assessed using the following trialwise measures: (1) trial duration as time from start to reward, (2) mean velocity, (3) immobility (velocity < 5 cm/s), and (4) navigation errors.

Errors were defined at intersections as choices that did not reduce the distance to the goal; a choice was locked in if the animal crossed a 20 cm-radius circle centered at each intersection. The maze contained no loops, i.e., there was only one correct choice at each intersection.

###  Electrophysiological recordings

Recordings were obtained using a Digital Lynx acquisition system (Neuralynx, Bozeman, Montana, USA.) with a 32-channel headstage and custom adapter (SSD-10-SS-GS, Omnetic). A skull screw above the cerebellum served as the electrode reference and the ground. Broadband signals (1–9,000 Hz) were digitized at 32 kHz.

Following the final recording session, rats were euthanized (Narcoren, 100 mg/kg i.p., Merial, Lyon, France.) and transcardially perfused with 0.9% saline followed by 4% paraformaldehyde. Brains were sectioned horizontally (50 μm; Microm HM 440E, Thermo Fischer Scientific, Waltham, Massachusetts, USA.), and electrode placements were manually verified in Nissl-stained sections. Representative sections showing electrode tracks and placement within RE are provided in Supplementary Figure S1.

###  Signal processing

Signals were detrended (1 s windows, 0.5 s overlap) using locdeterend (Chronux, Matlab), artifact-cleaned using custom Matlab scripts based on Islam et al. (2014), and downsampled to 1 kHz after applying a zero-phase-delay FIR anti-aliasing low-pass filter. Line noise was removed using notch filters at 50 and 100 Hz. Trials with residual artifacts were excluded based on visual inspection of their power spectra (42 trials, 10.0%).

###  Spectral and connectivity analysis

All post-processing analyses and statistics were performed in Python using custom scripts. Power spectral density (PSD) was estimated using Welch's method (1 s windows, 0.5 s overlap), not log-transformed, and not 1/f-corrected. Functional connectivity was estimated for each pair of recording sites (PFC-RE, RE-HC, HC-PFC) using coherence and Granger causality (GC). Coherence and GC were computed from time-frequency representations derived from Morlet wavelets (six cycles, 5–130 Hz, log-spaced frequencies), as implemented in the mne-connectivity Python library (Binns et al., 2026). All measures were estimated across full trials. For model fits and statistical comparisons, results were averaged within predefined frequency bands based primarily on conventional frequency ranges used in the literature: theta (6–11 Hz), beta (15–30 Hz), low gamma (30–60 Hz), and high gamma (60–120 Hz). The theta and beta ranges additionally encompassed the prominent spectral peaks observed in the present data.

The weighted phase-lag index (wPLI) and time-reversed Granger causality (tr-GC) were used to reduce the chance of false positives due to noise. The measures were computed in the same way as coherence and GC (mne-connectivity).

###  Statistical analysis

To assess learning-related effects while controlling for velocity and repeated measures, linear mixed-effects models (statsmodels, Python) were fit to each measure (band-averaged PSD, coherence, GC) across all trials, with trial-averaged velocity as a covariate:

measure ~ C(day) + velocity + (1|rat).

Trial-averaged velocity was included as a covariate to statistically control for movement-related modulation of neural activity, ensuring that estimated effects of day reflected learning-related changes beyond those explained by behavioral velocity differences. Day was treated as a categorical variable C(day) to avoid assuming monotonicity. The existence of a learning effect was estimated by comparing the models to reduced models of the form

measure ~ velocity + (1|rat)

using likelihood-ratio tests. The time course and direction of learning-related changes were inferred directly from the model coefficients for each day. To control for multiple comparisons across tests, we applied a False Discovery Rate (FDR) correction using the Holm-Bonferroni procedure to all test results. The significance threshold was set at an FDR of α = 0.05.

To compare forward and time-reversed GC, we fit linear mixed-effects models without a velocity covariate, i.e.,

tr-GC ~ C(day) + (1|rat),

to avoid fitting velocity to separate parts of the signal for forward and time-reversed measures.

## Data Availability

The raw data supporting the conclusions of this article will be made available by the authors, without undue reservation.
