# Supplementary material for: Spatial learning strengthens hippocampal but not prefrontal influence on nucleus reuniens in rats
Source: Front Behav Neurosci. 2026 Sep 25;20:1939794. doi: 10.3389/fnbeh.2026.1939794 (PMC13649685; doi:10.3389/fnbeh.2026.1939794)
Supplement: Supplementary file 1 [file Data_Sheet_1.pdf]

Supplementary figures

Rat ID	AP from Bregma (mm)	Rat ID	AP from Bregma (mm)	Rat ID	AP from Bregma (mm)
829.1	RE: -2.04	835.1	RE: -1.8mm (central RE)	856.2	RE: -2.04 (central RE)
829.2	RE: -1.56 (ventral RE)	856.1	RE: -2.28 (central RE)	861.1	RE: -1.92

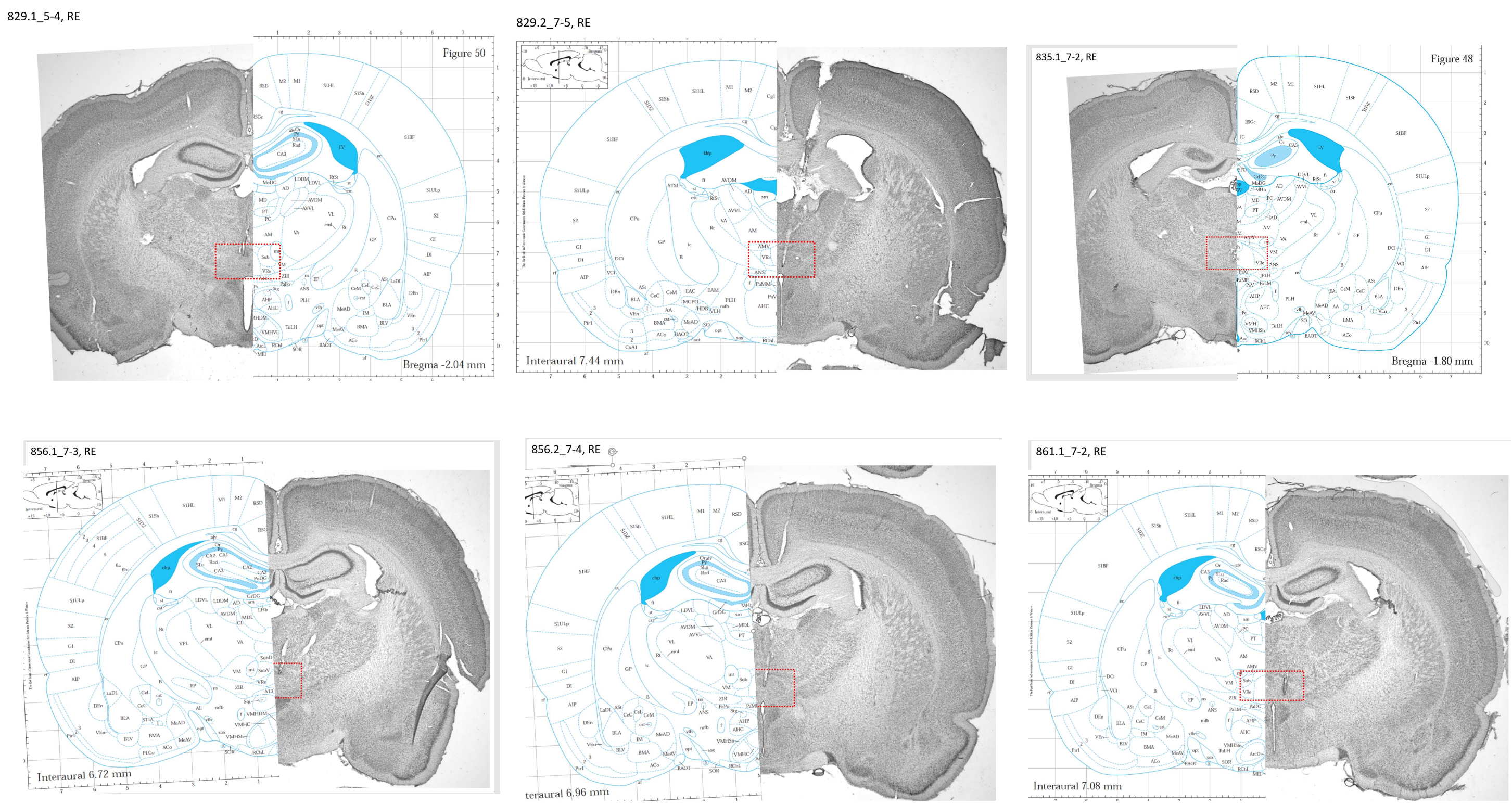

Supplementary Figure 1. Histological verification of RE electrode placement. Representative sections from each rat (corresponding to R1-6, in ascending order) showing the electrode track and recording location within RE, coregistered to the rat brain atlas (***Paxinos and Watson, 2006***).

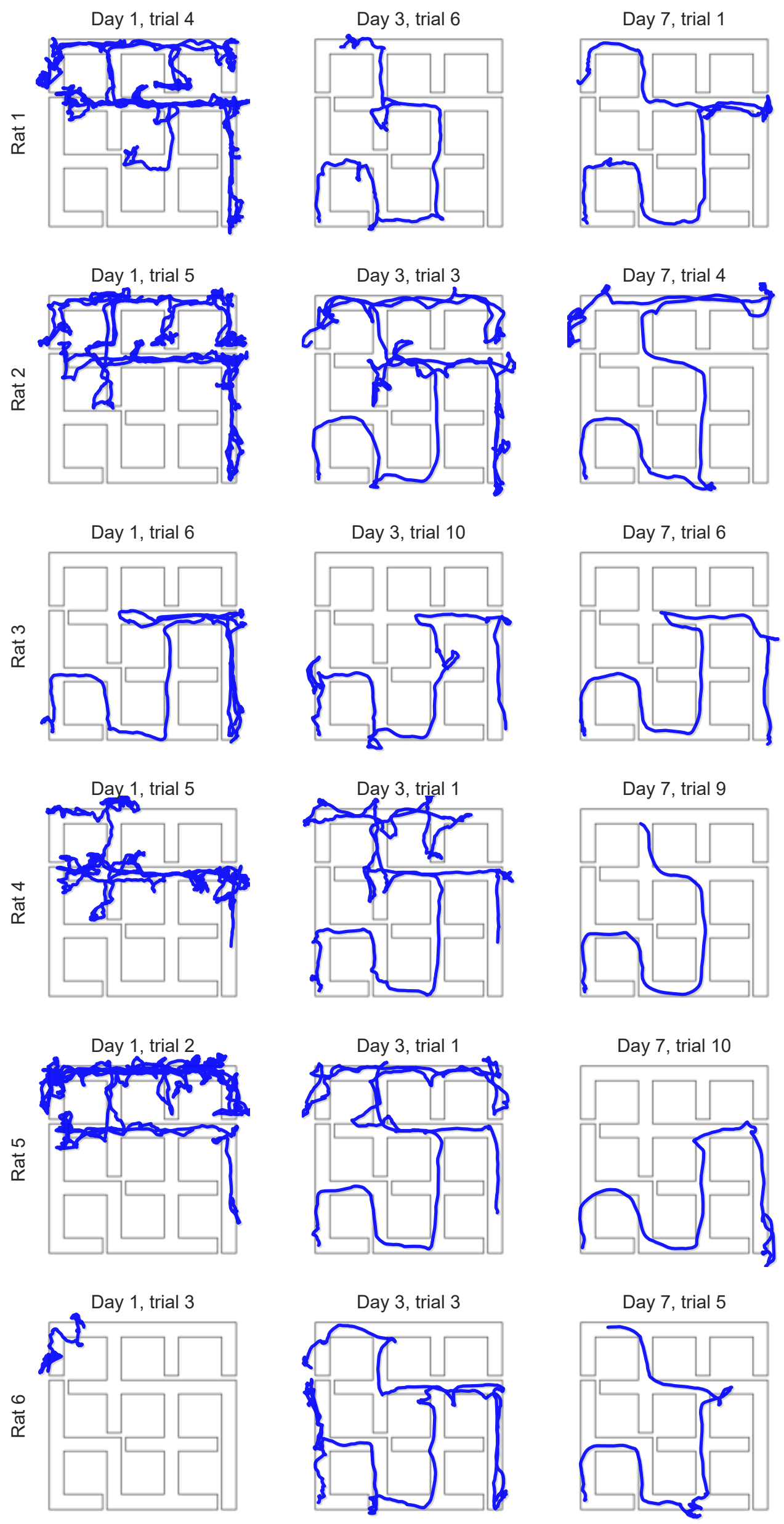

Supplementary Figure 2. Example trajectories (randomly selected trials) for each rat, days 1, 3, and 7.

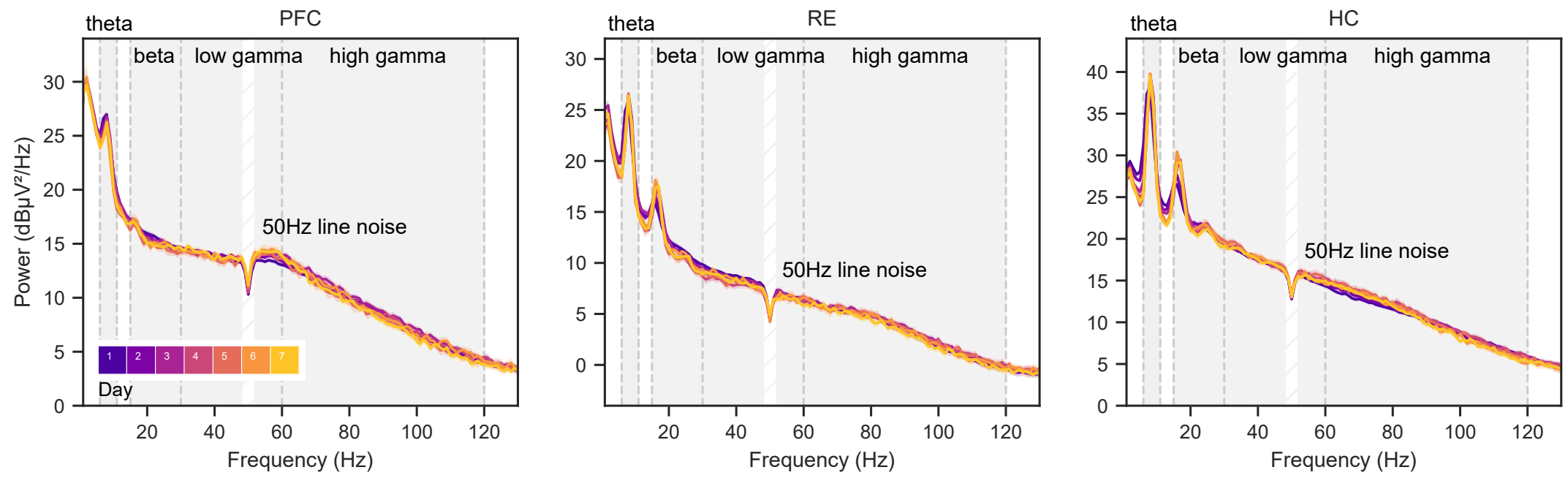

Supplementary Figure 3. Power spectral density across days (color gradient). All regions show a clear theta peak at ∼8Hz, as well as a second, lower, beta peak at ∼16Hz. The sharp depression at 50Hz is a filtering artifact resulting from 50Hz line noise removal (notch filter). Band power was estimated by averaging power across predefined ranges (gray bars); the plot shows log-transformed power for visibility, but untransformed values were used for model estimates.

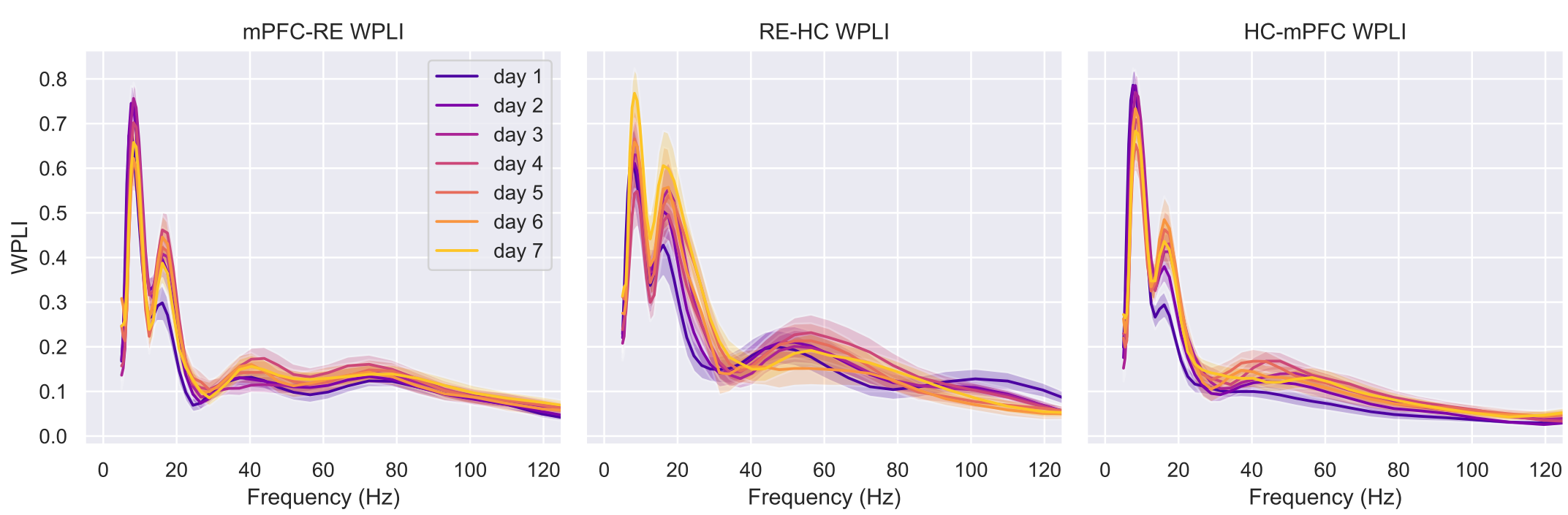

Supplementary Figure 4. Weighted phase-lag index (WPLI) across days (color gradient). Shading indicates 95% CI.

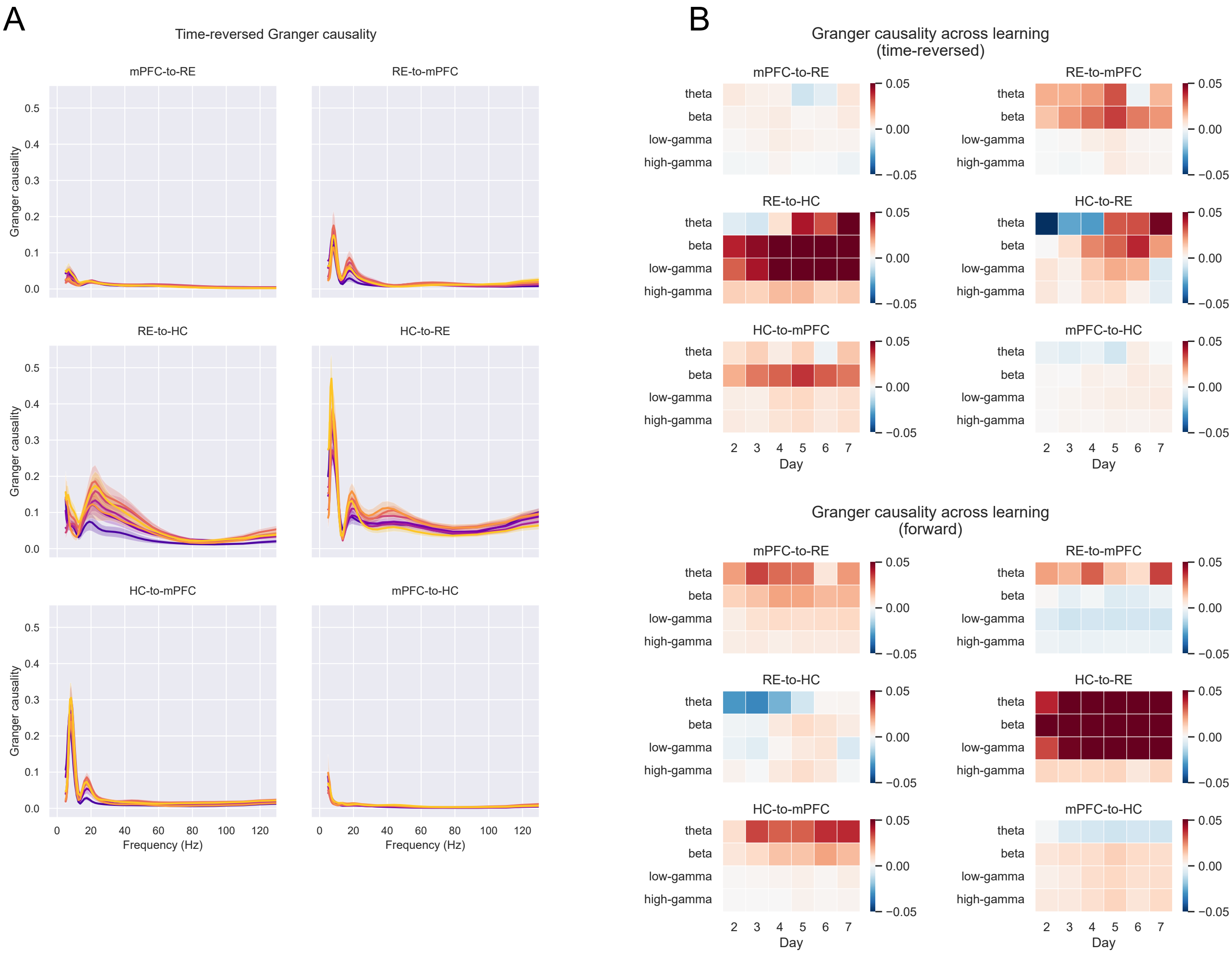

Supplementary Figure 5. Time-reversed Granger causality (tr-GC). True positives should reverse for tr-GC, i.e., show up in the opposite direction. **(a)** Tr-GC across days (color gradient). Shading indicates 95% CI. HC-to-RE beta and low-gamma GC reversed, as indicated by the increase in RE-to-HC tr-GC. **(b)** Coefficient plot of tr-GC (top) and forward GC (bottom). The linear model used here did not include velocity as a covariate to avoid fitting it to separate components of the forward and time-reversed signals.

Supplementary tables

LME comparison and coefficients, power

region	band	term	lr_stat	df_diff	p_unc	p_corr
PFC	theta	lrt	52.555	6.000	0.000	0.000
	beta	lrt	19.194	6.000	0.004	0.004
	low_gamma	lrt	58.327	6.000	0.000	0.000
	high_gamma	lrt	93.981	6.000	0.000	0.000
RE	theta	lrt	44.398	6.000	0.000	0.000
	beta	lrt	44.929	6.000	0.000	0.000
	low_gamma	lrt	34.148	6.000	0.000	0.000
	high_gamma	lrt	31.144	6.000	0.000	0.000
HC	theta	lrt	53.425	6.000	0.000	0.000
	beta	lrt	35.054	6.000	0.000	0.000
	low_gamma	lrt	94.019	6.000	0.000	0.000
	high_gamma	lrt	46.160	6.000	0.000	0.000

Supplementary Table 1. LME model comparison (likelihood-ratio-test). Full model: `power ~ velocity + C(day) + (1|rat)`. Reduced model: `power ~ velocity + (1|rat)`.

region	band	term	coef	se	z	p_unc	p_corr
PFC	theta	Intercept	348.345	20.631	16.885	0.000	0.000
		C(day)[T.2]	-25.118	10.399	-2.415	0.016	0.027
		C(day)[T.3]	-30.738	11.659	-2.636	0.008	0.015
		C(day)[T.4]	-62.076	13.280	-4.674	0.000	0.000
		C(day)[T.5]	-85.001	14.163	-6.002	0.000	0.000
		C(day)[T.6]	-89.438	15.066	-5.936	0.000	0.000
		C(day)[T.7]	-92.272	14.973	-6.163	0.000	0.000
		v	0.750	0.477	1.573	0.116	0.136
		Group Var	0.698	0.416	1.679	0.093	0.115
	beta	Intercept	38.602	1.700	22.701	0.000	0.000
		C(day)[T.2]	-0.471	0.994	-0.474	0.636	0.648
		C(day)[T.3]	-0.732	1.116	-0.656	0.512	0.535
		C(day)[T.4]	-2.814	1.274	-2.209	0.027	0.043
		C(day)[T.5]	-4.977	1.359	-3.662	0.000	0.001
		C(day)[T.6]	-2.569	1.447	-1.776	0.076	0.106
		C(day)[T.7]	-2.861	1.437	-1.992	0.046	0.070
		v	0.028	0.046	0.620	0.535	0.551
		Group Var	0.467	0.284	1.643	0.100	0.122
	low_gamma	Intercept	23.670	1.049	22.567	0.000	0.000
		C(day)[T.2]	1.748	0.373	4.689	0.000	0.000
		C(day)[T.3]	2.708	0.419	6.460	0.000	0.000
		C(day)[T.4]	2.481	0.478	5.194	0.000	0.000
		C(day)[T.5]	2.710	0.510	5.315	0.000	0.000
		C(day)[T.6]	2.692	0.543	4.956	0.000	0.000
		C(day)[T.7]	4.084	0.540	7.559	0.000	0.000
		v	-0.043	0.017	-2.458	0.014	0.025
		Group Var	1.605	0.951	1.687	0.092	0.115
	high_gamma	Intercept	8.178	0.658	12.430	0.000	0.000
		C(day)[T.2]	0.153	0.162	0.944	0.345	0.373
		C(day)[T.3]	0.901	0.182	4.959	0.000	0.000
		C(day)[T.4]	0.240	0.207	1.157	0.247	0.278
		C(day)[T.5]	-0.435	0.221	-1.967	0.049	0.073
		C(day)[T.6]	-0.472	0.235	-2.007	0.045	0.068
		C(day)[T.7]	-0.523	0.234	-2.233	0.026	0.041
		v	0.033	0.007	4.397	0.000	0.000
		Group Var	3.576	2.097	1.705	0.088	0.115

Supplementary Table 2. LME coefficients, PFC power

region	band	term	coef	se	z	p_unc	p_corr
RE	theta	Intercept	216.218	18.241	11.853	0.000	0.000
		C(day)[T.2]	-21.544	9.118	-2.363	0.018	0.031
		C(day)[T.3]	-24.874	10.226	-2.432	0.015	0.026
		C(day)[T.4]	-65.771	11.654	-5.644	0.000	0.000
		C(day)[T.5]	-60.543	12.431	-4.870	0.000	0.000
		C(day)[T.6]	-70.297	13.218	-5.318	0.000	0.000
		C(day)[T.7]	-71.013	13.143	-5.403	0.000	0.000
		v	1.720	0.419	4.104	0.000	0.000
		Group Var	0.713	0.425	1.678	0.093	0.115
	beta	Intercept	15.629	2.383	6.560	0.000	0.000
		C(day)[T.2]	-0.939	0.751	-1.251	0.211	0.242
		C(day)[T.3]	-0.673	0.843	-0.799	0.425	0.454
		C(day)[T.4]	-5.112	0.960	-5.324	0.000	0.000
		C(day)[T.5]	-4.533	1.024	-4.425	0.000	0.000
		C(day)[T.6]	-3.669	1.090	-3.366	0.001	0.002
		C(day)[T.7]	-4.024	1.084	-3.711	0.000	0.000
		v	0.328	0.035	9.483	0.000	0.000
		Group Var	2.097	1.230	1.705	0.088	0.115
	low_gamma	Intercept	7.154	0.248	28.892	0.000	0.000
		C(day)[T.2]	-0.021	0.118	-0.177	0.859	0.867
		C(day)[T.3]	0.389	0.132	2.941	0.003	0.006
		C(day)[T.4]	0.009	0.151	0.060	0.952	0.952
		C(day)[T.5]	0.394	0.161	2.454	0.014	0.025
		C(day)[T.6]	0.401	0.171	2.344	0.019	0.032
		C(day)[T.7]	0.639	0.170	3.756	0.000	0.000
		v	-0.043	0.005	-7.961	0.000	0.000
		Group Var	0.805	0.478	1.683	0.092	0.115
	high_gamma	Intercept	2.318	0.148	15.707	0.000	0.000
		C(day)[T.2]	-0.051	0.047	-1.083	0.279	0.310
		C(day)[T.3]	0.055	0.052	1.054	0.292	0.322
		C(day)[T.4]	-0.171	0.060	-2.859	0.004	0.008
		C(day)[T.5]	-0.146	0.064	-2.286	0.022	0.036
		C(day)[T.6]	-0.142	0.068	-2.100	0.036	0.056
		C(day)[T.7]	-0.188	0.068	-2.784	0.005	0.010
		v	0.008	0.002	3.512	0.000	0.001
		Group Var	2.082	1.231	1.692	0.091	0.115

Supplementary Table 3. LME coefficients, RE power

region	band	term	coef	se	z	p_unc	p_corr
HC	theta	Intercept	3277.721	413.343	7.930	0.000	0.000
		C(day)[T.2]	-225.235	140.815	-1.600	0.110	0.132
		C(day)[T.3]	-152.581	158.159	-0.965	0.335	0.365
		C(day)[T.4]	-561.815	180.168	-3.118	0.002	0.004
		C(day)[T.5]	-819.758	192.256	-4.264	0.000	0.000
		C(day)[T.6]	-1098.185	204.619	-5.367	0.000	0.000
		C(day)[T.7]	-1132.309	203.565	-5.562	0.000	0.000
		v	42.230	6.504	6.493	0.000	0.000
		Group Var	1.765	1.039	1.698	0.090	0.115
	beta	Intercept	149.214	18.540	8.048	0.000	0.000
		C(day)[T.2]	13.713	8.623	1.590	0.112	0.133
		C(day)[T.3]	34.815	9.672	3.600	0.000	0.001
		C(day)[T.4]	20.320	11.019	1.844	0.065	0.095
		C(day)[T.5]	24.044	11.754	2.046	0.041	0.063
		C(day)[T.6]	14.765	12.506	1.181	0.238	0.270
		C(day)[T.7]	-8.383	12.429	-0.674	0.500	0.529
		v	4.066	0.396	10.258	0.000	0.000
		Group Var	0.855	0.507	1.687	0.092	0.115
	low_gamma	Intercept	54.154	4.678	11.576	0.000	0.000
		C(day)[T.2]	3.880	1.079	3.596	0.000	0.001
		C(day)[T.3]	8.801	1.212	7.261	0.000	0.000
		C(day)[T.4]	9.564	1.381	6.926	0.000	0.000
		C(day)[T.5]	14.334	1.474	9.726	0.000	0.000
		C(day)[T.6]	13.625	1.569	8.685	0.000	0.000
		C(day)[T.7]	12.181	1.561	7.804	0.000	0.000
		v	-0.340	0.050	-6.813	0.000	0.000
		Group Var	4.087	2.393	1.708	0.088	0.115
	high_gamma	Intercept	11.259	1.001	11.251	0.000	0.000
		C(day)[T.2]	0.376	0.272	1.382	0.167	0.194
		C(day)[T.3]	1.135	0.306	3.709	0.000	0.000
		C(day)[T.4]	1.189	0.349	3.411	0.001	0.001
		C(day)[T.5]	0.671	0.372	1.803	0.071	0.101
		C(day)[T.6]	0.715	0.396	1.806	0.071	0.101
		C(day)[T.7]	-0.256	0.394	-0.651	0.515	0.535
		v	0.072	0.013	5.702	0.000	0.000
		Group Var	2.881	1.691	1.703	0.088	0.115

Supplementary Table 4. LME coefficients, HC power

LME comparison and coefficients, coherence

region_i	region_j	band	term	lr_stat	df_diff	p_unc	p_corr
PFC	RE	theta	lrt	23.129	6.000	0.001	0.001
		beta	lrt	40.571	6.000	0.000	0.000
RE	HC	theta	lrt	9.262	6.000	0.159	0.191
		beta	lrt	51.697	6.000	0.000	0.000
HC	PFC	theta	lrt	5.232	6.000	0.514	0.514
		beta	lrt	22.755	6.000	0.001	0.001

Supplementary Table 5. LME model comparison (likelihood-ratio-test). Full model: `coherence ~ velocity + C(day) + (1|rat)`. Reduced model: `coherence ~ velocity + (1|rat)`.

region_i	region_j	band	term	coef	se	z	p_unc	p_corr
PFC	RE	theta	Intercept	0.563	0.024	23.168	0.000	0.000
			C(day)[T.2]	-0.017	0.014	-1.282	0.200	0.277
			C(day)[T.3]	-0.046	0.015	-3.003	0.003	0.007
			C(day)[T.4]	-0.042	0.017	-2.422	0.015	0.030
			C(day)[T.5]	-0.013	0.019	-0.680	0.496	0.609
			C(day)[T.6]	-0.034	0.020	-1.714	0.087	0.149
			C(day)[T.7]	-0.068	0.020	-3.460	0.001	0.002
			v	0.001	0.001	1.845	0.065	0.117
			Group Var	0.527	0.320	1.647	0.100	0.149
		beta	Intercept	0.465	0.018	25.247	0.000	0.000
			C(day)[T.2]	-0.034	0.010	-3.581	0.000	0.001
			C(day)[T.3]	-0.069	0.011	-6.425	0.000	0.000
			C(day)[T.4]	-0.052	0.012	-4.208	0.000	0.000
			C(day)[T.5]	-0.051	0.013	-3.875	0.000	0.000
			C(day)[T.6]	-0.045	0.014	-3.200	0.001	0.004
			C(day)[T.7]	-0.059	0.014	-4.272	0.000	0.000
			v	-0.002	0.000	-3.727	0.000	0.001
			Group Var	0.638	0.384	1.663	0.096	0.149

Supplementary Table 6. LME coefficients, RE-PFC coherence

region_i	region_j	band	term	coef	se	z	p_unc	p_corr
RE	HC	theta	Intercept	0.765	0.021	35.925	0.000	0.000
			C(day)[T.2]	0.006	0.015	0.386	0.700	0.738
			C(day)[T.3]	-0.021	0.016	-1.254	0.210	0.283
			C(day)[T.4]	-0.007	0.019	-0.384	0.701	0.738
			C(day)[T.5]	0.020	0.020	1.016	0.309	0.398
			C(day)[T.6]	-0.010	0.021	-0.455	0.649	0.738
			C(day)[T.7]	-0.009	0.021	-0.415	0.678	0.738
			v	-0.002	0.001	-2.870	0.004	0.009
			Group Var	0.279	0.173	1.614	0.106	0.155
		beta	Intercept	0.369	0.021	17.992	0.000	0.000
			C(day)[T.2]	0.030	0.010	3.111	0.002	0.005
			C(day)[T.3]	0.028	0.011	2.631	0.009	0.018
			C(day)[T.4]	0.012	0.012	0.965	0.335	0.420
			C(day)[T.5]	0.006	0.013	0.438	0.661	0.738
			C(day)[T.6]	-0.007	0.014	-0.525	0.600	0.720
			C(day)[T.7]	-0.035	0.014	-2.509	0.012	0.024
			v	0.006	0.000	14.134	0.000	0.000
			Group Var	0.841	0.501	1.680	0.093	0.149

Supplementary Table 7. LME coefficients, HC-RE coherence

region_i	region_j	band	term	coef	se	z	p_unc	p_corr
HC	PFC	theta	Intercept	0.516	0.032	15.966	0.000	0.000
			C(day)[T.2]	-0.005	0.014	-0.372	0.710	0.738
			C(day)[T.3]	-0.025	0.016	-1.590	0.112	0.159
			C(day)[T.4]	-0.020	0.018	-1.101	0.271	0.357
			C(day)[T.5]	-0.003	0.019	-0.132	0.895	0.895
			C(day)[T.6]	-0.005	0.020	-0.261	0.794	0.809
			C(day)[T.7]	-0.009	0.020	-0.431	0.666	0.738
			v	-0.002	0.001	-2.893	0.004	0.009
			Group Var	1.018	0.607	1.679	0.093	0.149
		beta	Intercept	0.113	0.014	7.893	0.000	0.000
			C(day)[T.2]	0.027	0.009	3.124	0.002	0.005
			C(day)[T.3]	0.029	0.010	2.979	0.003	0.007
			C(day)[T.4]	0.038	0.011	3.376	0.001	0.002
			C(day)[T.5]	0.048	0.012	4.041	0.000	0.000
			C(day)[T.6]	0.039	0.013	3.119	0.002	0.005
			C(day)[T.7]	0.025	0.013	1.974	0.048	0.090
			v	0.002	0.000	5.276	0.000	0.000
			Group Var	0.413	0.249	1.655	0.098	0.149

Supplementary Table 8. LME coefficients, HC-PFC coherence

LME comparison and coefficients, Granger causality

direction	band	term	lr_stat	df_diff	p_unc	p_corr
PFC-to-RE	theta	lrt	22.104	6.000	0.001	0.003
	beta	lrt	23.353	6.000	0.001	0.002
	low_gamma	lrt	13.893	6.000	0.031	0.046
	high_gamma	lrt	24.477	6.000	0.000	0.001
RE-to-PFC	theta	lrt	15.439	6.000	0.017	0.027
	beta	lrt	19.942	6.000	0.003	0.005
	low_gamma	lrt	27.923	6.000	0.000	0.001
	high_gamma	lrt	10.672	6.000	0.099	0.140
RE-to-HC	theta	lrt	40.506	6.000	0.000	0.000
	beta	lrt	7.743	6.000	0.258	0.309
	low_gamma	lrt	26.509	6.000	0.000	0.001
	high_gamma	lrt	19.325	6.000	0.004	0.006
HC-to-RE	theta	lrt	33.253	6.000	0.000	0.000
	beta	lrt	20.548	6.000	0.002	0.004
	low_gamma	lrt	31.056	6.000	0.000	0.000
	high_gamma	lrt	25.847	6.000	0.000	0.001
HC-to-PFC	theta	lrt	4.917	6.000	0.554	0.579
	beta	lrt	7.166	6.000	0.306	0.349
	low_gamma	lrt	10.187	6.000	0.117	0.156
	high_gamma	lrt	23.570	6.000	0.001	0.002
PFC-to-HC	theta	lrt	0.422	6.000	0.999	0.999
	beta	lrt	9.444	6.000	0.150	0.190
	low_gamma	lrt	5.001	6.000	0.544	0.579
	high_gamma	lrt	26.697	6.000	0.000	0.001

Supplementary Table 9. LME model comparison (likelihood-ratio-test). Full model: `GC ~ velocity + C(day) + (1 rat)`. Reduced model: `GC ~ velocity + (1 rat)`.

direction	band	term	coef	se	z	p_unc	p_corr
PFC-to-RE	theta	Intercept	0.037	0.014	2.597	0.009	0.031
		C(day)[T.2]	0.013	0.009	1.417	0.156	0.244
		C(day)[T.3]	0.009	0.011	0.885	0.376	0.497
		C(day)[T.4]	-0.004	0.012	-0.356	0.721	0.799
		C(day)[T.5]	-0.012	0.013	-0.943	0.346	0.464
		C(day)[T.6]	-0.035	0.014	-2.459	0.014	0.042
		C(day)[T.7]	-0.023	0.014	-1.695	0.090	0.177
		v	0.002	0.000	4.337	0.000	0.000
		Group Var	0.386	0.263	1.469	0.142	0.230
	beta	Intercept	-0.000	0.003	-0.019	0.985	0.989
		C(day)[T.2]	0.008	0.003	2.946	0.003	0.012
		C(day)[T.3]	0.003	0.003	1.150	0.250	0.365
		C(day)[T.4]	0.005	0.003	1.522	0.128	0.219
		C(day)[T.5]	0.002	0.004	0.512	0.609	0.707
		C(day)[T.6]	-0.003	0.004	-0.714	0.475	0.580
		C(day)[T.7]	-0.004	0.004	-0.960	0.337	0.455
		v	0.001	0.000	7.559	0.000	0.000
		Group Var	0.040	0.036	1.120	0.263	0.376
	low_gamma	Intercept	0.001	0.002	0.665	0.506	0.607
		C(day)[T.2]	0.003	0.001	2.329	0.020	0.056
		C(day)[T.3]	0.005	0.001	3.172	0.002	0.007
		C(day)[T.4]	0.006	0.002	3.370	0.001	0.004
		C(day)[T.5]	0.004	0.002	2.054	0.040	0.095
		C(day)[T.6]	0.004	0.002	2.181	0.029	0.075
		C(day)[T.7]	0.005	0.002	2.580	0.010	0.032
		v	0.000	0.000	3.701	0.000	0.001
		Group Var	0.397	0.272	1.460	0.144	0.232
	high_gamma	Intercept	0.004	0.004	1.033	0.302	0.420
		C(day)[T.2]	0.004	0.001	4.034	0.000	0.000
		C(day)[T.3]	0.005	0.001	4.426	0.000	0.000
		C(day)[T.4]	0.004	0.001	3.029	0.002	0.010
		C(day)[T.5]	0.005	0.001	3.960	0.000	0.001
		C(day)[T.6]	0.005	0.001	3.467	0.001	0.003
		C(day)[T.7]	0.005	0.001	3.955	0.000	0.001
		v	-0.000	0.000	-0.165	0.869	0.907
		Group Var	3.589	2.310	1.554	0.120	0.219

Supplementary Table 10. LME coefficients, PFC-to-RE GC

direction	band	term	coef	se	z	p_unc	p_corr
RE-to-PFC	theta	Intercept	0.058	0.019	3.054	0.002	0.009
		C(day)[T.2]	0.014	0.009	1.583	0.113	0.219
		C(day)[T.3]	-0.004	0.010	-0.394	0.694	0.779
		C(day)[T.4]	0.003	0.012	0.223	0.823	0.877
		C(day)[T.5]	-0.018	0.012	-1.438	0.151	0.239
		C(day)[T.6]	-0.025	0.014	-1.821	0.069	0.141
		C(day)[T.7]	-0.003	0.013	-0.244	0.807	0.871
		v	0.002	0.000	3.748	0.000	0.001
		Group Var	0.888	0.585	1.519	0.129	0.219
	beta	Intercept	0.022	0.004	5.873	0.000	0.000
		C(day)[T.2]	-0.000	0.002	-0.217	0.828	0.877
		C(day)[T.3]	-0.008	0.003	-2.866	0.004	0.015
		C(day)[T.4]	-0.005	0.003	-1.807	0.071	0.144
		C(day)[T.5]	-0.012	0.003	-3.593	0.000	0.002
		C(day)[T.6]	-0.011	0.004	-3.130	0.002	0.008
		C(day)[T.7]	-0.008	0.003	-2.394	0.017	0.049
		v	0.000	0.000	2.088	0.037	0.089
		Group Var	0.414	0.276	1.503	0.133	0.223
	low_gamma	Intercept	0.021	0.002	9.218	0.000	0.000
		C(day)[T.2]	-0.005	0.001	-3.869	0.000	0.001
		C(day)[T.3]	-0.008	0.002	-5.116	0.000	0.000
		C(day)[T.4]	-0.007	0.002	-3.586	0.000	0.002
		C(day)[T.5]	-0.007	0.002	-3.302	0.001	0.005
		C(day)[T.6]	-0.006	0.002	-2.723	0.006	0.022
		C(day)[T.7]	-0.007	0.002	-3.111	0.002	0.008
		v	-0.000	0.000	-2.134	0.033	0.081
		Group Var	0.417	0.279	1.492	0.136	0.223
	high_gamma	Intercept	0.012	0.001	8.016	0.000	0.000
		C(day)[T.2]	-0.002	0.001	-2.359	0.018	0.052
		C(day)[T.3]	-0.003	0.001	-2.497	0.013	0.038
		C(day)[T.4]	-0.002	0.001	-1.423	0.155	0.244
		C(day)[T.5]	-0.001	0.001	-1.010	0.313	0.433
		C(day)[T.6]	-0.001	0.001	-0.618	0.537	0.633
		C(day)[T.7]	-0.001	0.001	-0.873	0.383	0.499
		v	-0.000	0.000	-1.986	0.047	0.106
		Group Var	0.409	0.281	1.456	0.145	0.232

Supplementary Table 11. LME coefficients, RE-to-PFC GC

direction	band	term	coef	se	z	p_unc	p_corr
RE-to-HC	theta	Intercept	0.095	0.026	3.649	0.000	0.002
		C(day)[T.2]	-0.034	0.008	-4.364	0.000	0.000
		C(day)[T.3]	-0.050	0.009	-5.444	0.000	0.000
		C(day)[T.4]	-0.046	0.010	-4.436	0.000	0.000
		C(day)[T.5]	-0.036	0.011	-3.228	0.001	0.006
		C(day)[T.6]	-0.028	0.012	-2.242	0.025	0.067
		C(day)[T.7]	-0.030	0.012	-2.508	0.012	0.037
		v	0.001	0.000	3.446	0.001	0.003
		Group Var	2.325	1.500	1.550	0.121	0.219
	beta	Intercept	0.041	0.010	4.058	0.000	0.000
		C(day)[T.2]	-0.005	0.004	-1.135	0.257	0.372
		C(day)[T.3]	-0.010	0.005	-2.158	0.031	0.078
		C(day)[T.4]	-0.005	0.005	-0.965	0.334	0.454
		C(day)[T.5]	-0.002	0.006	-0.400	0.689	0.779
		C(day)[T.6]	-0.005	0.006	-0.838	0.402	0.520
		C(day)[T.7]	-0.009	0.006	-1.556	0.120	0.219
		v	0.001	0.000	2.962	0.003	0.012
		Group Var	1.229	0.794	1.549	0.121	0.219
	low_gamma	Intercept	0.041	0.007	6.046	0.000	0.000
		C(day)[T.2]	-0.003	0.003	-1.064	0.287	0.403
		C(day)[T.3]	-0.007	0.004	-1.782	0.075	0.150
		C(day)[T.4]	-0.000	0.004	-0.022	0.982	0.989
		C(day)[T.5]	0.004	0.005	0.791	0.429	0.538
		C(day)[T.6]	0.005	0.005	1.100	0.271	0.386
		C(day)[T.7]	-0.009	0.005	-1.894	0.058	0.126
		v	0.000	0.000	0.427	0.669	0.769
		Group Var	0.836	0.549	1.521	0.128	0.219
	high_gamma	Intercept	0.036	0.008	4.782	0.000	0.000
		C(day)[T.2]	0.003	0.003	0.979	0.328	0.448
		C(day)[T.3]	0.001	0.003	0.278	0.781	0.848
		C(day)[T.4]	0.007	0.004	1.864	0.062	0.131
		C(day)[T.5]	0.011	0.004	2.853	0.004	0.015
		C(day)[T.6]	0.008	0.004	1.870	0.062	0.131
		C(day)[T.7]	0.002	0.004	0.390	0.696	0.779
		v	-0.000	0.000	-0.871	0.384	0.499
		Group Var	1.535	0.994	1.545	0.122	0.219

Supplementary Table 12. LME coefficients, RE-to-HC GC

direction	band	term	coef	se	z	p_unc	p_corr
HC-to-RE	theta	Intercept	0.146	0.050	2.938	0.003	0.012
		C(day)[T.2]	0.026	0.015	1.795	0.073	0.147
		C(day)[T.3]	0.037	0.017	2.205	0.027	0.072
		C(day)[T.4]	0.054	0.019	2.792	0.005	0.018
		C(day)[T.5]	0.114	0.021	5.469	0.000	0.000
		C(day)[T.6]	0.081	0.023	3.526	0.000	0.002
		C(day)[T.7]	0.081	0.022	3.695	0.000	0.001
		v	0.003	0.001	4.163	0.000	0.000
		Group Var	2.503	1.615	1.549	0.121	0.219
	beta	Intercept	0.041	0.022	1.855	0.064	0.132
		C(day)[T.2]	0.040	0.010	3.948	0.000	0.001
		C(day)[T.3]	0.033	0.012	2.850	0.004	0.015
		C(day)[T.4]	0.035	0.013	2.665	0.008	0.026
		C(day)[T.5]	0.050	0.014	3.509	0.000	0.002
		C(day)[T.6]	0.030	0.016	1.917	0.055	0.120
		C(day)[T.7]	0.033	0.015	2.170	0.030	0.076
		v	0.004	0.000	8.223	0.000	0.000
		Group Var	0.962	0.628	1.531	0.126	0.219
	low_gamma	Intercept	0.015	0.012	1.273	0.203	0.300
		C(day)[T.2]	0.021	0.007	3.209	0.001	0.006
		C(day)[T.3]	0.010	0.008	1.308	0.191	0.287
		C(day)[T.4]	0.018	0.009	1.995	0.046	0.106
		C(day)[T.5]	0.015	0.009	1.620	0.105	0.205
		C(day)[T.6]	-0.008	0.010	-0.759	0.448	0.553
		C(day)[T.7]	-0.006	0.010	-0.625	0.532	0.631
		v	0.003	0.000	9.136	0.000	0.000
		Group Var	0.555	0.371	1.496	0.135	0.223
	high_gamma	Intercept	0.014	0.005	3.059	0.002	0.009
		C(day)[T.2]	0.008	0.002	3.263	0.001	0.005
		C(day)[T.3]	0.002	0.003	0.814	0.416	0.530
		C(day)[T.4]	0.000	0.003	0.029	0.977	0.989
		C(day)[T.5]	-0.001	0.004	-0.320	0.749	0.817
		C(day)[T.6]	-0.007	0.004	-1.864	0.062	0.131
		C(day)[T.7]	-0.003	0.004	-0.931	0.352	0.469
		v	0.001	0.000	4.976	0.000	0.000
		Group Var	0.632	0.414	1.527	0.127	0.219

Supplementary Table 13. LME coefficients, HC-to-RE GC

direction	band	term	coef	se	z	p_unc	p_corr
HC-to-PFC	theta	Intercept	0.088	0.028	3.092	0.002	0.008
		C(day)[T.2]	-0.001	0.010	-0.114	0.909	0.931
		C(day)[T.3]	0.004	0.011	0.384	0.701	0.781
		C(day)[T.4]	-0.009	0.013	-0.703	0.482	0.585
		C(day)[T.5]	-0.016	0.014	-1.157	0.247	0.363
		C(day)[T.6]	-0.012	0.015	-0.783	0.433	0.538
		C(day)[T.7]	-0.016	0.015	-1.066	0.286	0.403
		v	0.002	0.000	4.811	0.000	0.000
		Group Var	1.801	1.168	1.541	0.123	0.219
	beta	Intercept	0.006	0.003	2.069	0.039	0.093
		C(day)[T.2]	0.003	0.003	1.350	0.177	0.270
		C(day)[T.3]	0.002	0.003	0.649	0.516	0.616
		C(day)[T.4]	0.005	0.003	1.415	0.157	0.244
		C(day)[T.5]	0.002	0.004	0.681	0.496	0.599
		C(day)[T.6]	0.008	0.004	2.007	0.045	0.105
		C(day)[T.7]	0.002	0.004	0.603	0.547	0.642
		v	0.001	0.000	4.924	0.000	0.000
		Group Var	0.100	0.075	1.332	0.183	0.276
	low_gamma	Intercept	0.002	0.001	2.521	0.012	0.037
		C(day)[T.2]	-0.000	0.001	-0.165	0.869	0.907
		C(day)[T.3]	-0.002	0.001	-1.564	0.118	0.219
		C(day)[T.4]	-0.001	0.001	-1.301	0.193	0.288
		C(day)[T.5]	-0.001	0.001	-0.883	0.377	0.497
		C(day)[T.6]	-0.003	0.001	-2.386	0.017	0.049
		C(day)[T.7]	-0.000	0.001	-0.342	0.732	0.806
		v	0.000	0.000	5.331	0.000	0.000
		Group Var	0.011	0.028	0.403	0.687	0.779
	high_gamma	Intercept	0.001	0.001	1.990	0.047	0.106
		C(day)[T.2]	0.000	0.000	0.528	0.598	0.698
		C(day)[T.3]	-0.000	0.000	-0.000	1.000	1.000
		C(day)[T.4]	-0.000	0.001	-0.094	0.925	0.943
		C(day)[T.5]	0.002	0.001	2.763	0.006	0.020
		C(day)[T.6]	0.000	0.001	0.736	0.462	0.567
		C(day)[T.7]	0.001	0.001	2.289	0.022	0.060
		v	0.000	0.000	0.811	0.418	0.530
		Group Var	0.441	0.296	1.489	0.136	0.223

Supplementary Table 14. LME coefficients, HC-to-PFC GC

direction	band	term	coef	se	z	p_unc	p_corr
PFC-to-HC	theta	Intercept	0.094	0.014	6.621	0.000	0.000
		C(day)[T.2]	0.001	0.006	0.217	0.829	0.877
		C(day)[T.3]	0.001	0.007	0.115	0.909	0.931
		C(day)[T.4]	0.002	0.009	0.210	0.833	0.878
		C(day)[T.5]	0.002	0.009	0.229	0.819	0.877
		C(day)[T.6]	0.004	0.010	0.415	0.678	0.775
		C(day)[T.7]	0.005	0.010	0.480	0.631	0.729
		v	-0.001	0.000	-1.989	0.047	0.106
		Group Var	0.926	0.608	1.524	0.128	0.219
	beta	Intercept	0.012	0.004	2.938	0.003	0.012
		C(day)[T.2]	0.005	0.002	2.280	0.023	0.061
		C(day)[T.3]	0.005	0.003	1.946	0.052	0.114
		C(day)[T.4]	0.005	0.003	1.701	0.089	0.176
		C(day)[T.5]	0.008	0.003	2.304	0.021	0.059
		C(day)[T.6]	0.005	0.004	1.349	0.177	0.270
		C(day)[T.7]	0.003	0.003	1.003	0.316	0.435
		v	0.000	0.000	1.981	0.048	0.106
		Group Var	0.514	0.340	1.512	0.131	0.220
	low_gamma	Intercept	0.005	0.002	2.131	0.033	0.081
		C(day)[T.2]	0.001	0.002	0.792	0.428	0.538
		C(day)[T.3]	-0.001	0.002	-0.338	0.735	0.806
		C(day)[T.4]	0.002	0.002	0.783	0.434	0.538
		C(day)[T.5]	0.002	0.003	0.826	0.409	0.526
		C(day)[T.6]	-0.000	0.003	-0.154	0.878	0.907
		C(day)[T.7]	-0.000	0.003	-0.159	0.873	0.907
		v	0.000	0.000	4.505	0.000	0.000
		Group Var	0.196	0.141	1.389	0.165	0.254
	high_gamma	Intercept	0.008	0.003	2.398	0.016	0.049
		C(day)[T.2]	0.005	0.001	3.161	0.002	0.007
		C(day)[T.3]	0.004	0.002	2.523	0.012	0.037
		C(day)[T.4]	0.007	0.002	3.511	0.000	0.002
		C(day)[T.5]	0.010	0.002	4.660	0.000	0.000
		C(day)[T.6]	0.005	0.002	2.204	0.028	0.072
		C(day)[T.7]	0.008	0.002	3.822	0.000	0.001
		v	0.000	0.000	1.125	0.261	0.376
		Group Var	1.189	0.777	1.530	0.126	0.219

Supplementary Table 15. LME coefficients, PFC-to-HC GC
